# Vipie: web pipeline for parallel characterization of viral populations from multiple NGS samples

**DOI:** 10.1186/s12864-017-3721-7

**Published:** 2017-05-15

**Authors:** Jake Lin, Lenka Kramna, Reija Autio, Heikki Hyöty, Matti Nykter, Ondrej Cinek

**Affiliations:** 1 BioMediTech and Faculty of Medicine and Life Sciences, University of Tampere, PB 100, FI-33014 Tampere, Finland; 2 Department of Pediatrics, 2nd Faculty of Medicine, Charles University and University Hospital Motol, V Úvalu 84, 150 06 Praha 5, Czech Republic; 3 School of Social Sciences, University of Tampere, Kalevantie 4, 33100 Tampere, Finland; 4 Fimlab Laboratories, Pirkanmaa Hospital District, Tampere, Finland

**Keywords:** *Metagenomics*, Viromes, Virus, Assembly, NGS analysis, Visualization, Parallel processing, Viral dark matter

## Abstract

**Background:**

Next generation sequencing (NGS) technology allows laboratories to investigate virome composition in clinical and environmental samples in a culture-independent way. There is a need for bioinformatic tools capable of parallel processing of virome sequencing data by exactly identical methods: this is especially important in studies of multifactorial diseases, or in parallel comparison of laboratory protocols.

**Results:**

We have developed a web-based application allowing direct upload of sequences from multiple virome samples using custom parameters. The samples are then processed in parallel using an identical protocol, and can be easily reanalyzed. The pipeline performs de-novo assembly, taxonomic classification of viruses as well as sample analyses based on user-defined grouping categories. Tables of virus abundance are produced from cross-validation by remapping the sequencing reads to a union of all observed reference viruses. In addition, read sets and reports are created after processing unmapped reads against known human and bacterial ribosome references. Secured interactive results are dynamically plotted with population and diversity charts, clustered heatmaps and a sortable and searchable abundance table.

**Conclusions:**

The Vipie web application is a unique tool for multi-sample metagenomic analysis of viral data, producing searchable hits tables, interactive population maps, alpha diversity measures and clustered heatmaps that are grouped in applicable custom sample categories. Known references such as human genome and bacterial ribosomal genes are optionally removed from unmapped (‘dark matter’) reads. Secured results are accessible and shareable on modern browsers. Vipie is a freely available web-based tool whose code is open source.

**Electronic supplementary material:**

The online version of this article (doi:10.1186/s12864-017-3721-7) contains supplementary material, which is available to authorized users.

## Background

The use of virome metagenomics has been growing rapidly due to the increasing demands to study the whole virome in clinical samples and to evaluate the evolution of viral quasispecies during acute and chronic infections. The application of virome sequencing techniques become useful not only in infectious disease research, but also in association studies of primarily non-infectious conditions, i.e. in diseases where the agent is presumed to modify the risk of the disease, which effect is detectable upon investigation of a large number of subjects only. These applications require an approximation of virus quantity, similar to what has long been utilized in bacteriome profiling.

As viruses lack a common sequence signature, metagenomics sequencing of random viral libraries remains the only feasible way of an unbiased assessment of the whole virome. Presently, the need for accurate quantification and interpretation of viral population metrics across a set of samples creates a substantial challenge for this kind of metagenomics studies. Prime obstacles for virome investigators are the large genetic heterogeneity and also that the majority of bioinformatic tools are command line based and overtly technical, being computationally demanding, with complicated dependencies, and producing text based outputs that are not easily interpretable [1–5]. Recently released web based applications Taxonomer [6], VirusTAP [7], Virome [8] and Metavir [9, 10] have addressed some of the issues (especially those of user interaction), but mostly operate only on single sample experiments with different workflows. Requiring local dependencies and installation, ViromeScan [11] and MetaShot [12] works on multiple samples. Some of these tools were designed for long (>300) reads or assembled contigs [8–10], which is limiting as modern metagenomics projects including Human Microbiome Project (HMP) [1, 2] produce mostly high-throughput short paired reads. Table 1 provides an overview of the primary features and strategies of these different tools, including our work.

**Table 1:** Comparison of the existing virome pipelines tools

Pipeline Tool	Vipie	ViromeScan [11]	VirusTAP [8]	Virome [16]	Metavir [14]	Taxonomer [6]	MetaShot [12]
Primary goal	Parallel analysis of multiple viral metagenomes from web and suited for molecular epidemiology studies.	To profile viromes using databases of existing eukaryotic viruses without assembly.	Identification of viruses in a sample, after a thorough elimination of known non-viral sequences.	Classification of all putative ORF found in a viral metagenome, characterization of viral communities.	Analysis of virome, diversity metrics and marker gene phylogenies.	Ultra fast metagenomics analysis focusing on detection of microorganisms, including virus and bacterial.	Highly accurate and comprehensive workflow for host-associate microbiome classification on multiple samples.
Web based	Yes.	No.	Yes.	Yes (Flash required).	Yes.	Yes.	No.
Outputs	Interactive table, plots and raw downloads. Clustered heatmaps with dynamic group assignment re-plots.	Static population pie charts. Sample based clustered heatmaps.	Contig based hits and seamless web BLAST interface.	Rich collection of sample source virome ORF and sequence categories.	Comparative analysis of viromes and annotations including networks, nonmetric distance and tree maps.	Interactive pie charts with kingdoms in bins and also impressive sunburst flare sub classifiers.	A Krona graph and Interactive Taxonomy HTML table along with csv file.
Source data	Paired-end reads; *fastq* format.	Sinle-end or paired-end reads; *fastq* format.	Paired-end reads. Accepts also single-end reads; *fastq* format.	*sff,* or *fastq*; intended for the 454-generated metagenomes.	Reads (>300 bases) or assembled contigs.	Paired-end reads in fastq and fasta formats.	Paired-end reads in fastq format.
Trimming and filtering	YES, as the first step.	YES, after selection of viral reads, at the level of a *bam* file.	YES, as the first step.	YES: quality based; duplicate filtering; contamination	Not specified.	Not specified.	YES, as the first step.
De-novo assembly	YES, a choice of assemblers.	No.	YES, a choice of assemblers; done after subtraction steps.	No.	No.	No.	No.
Subtraction of human ref. and bacterial ribosomal sequences	Optional, only for the output of dark matter sequences.	YES, using Human Best Match Tagger. No for ribosomal.	YES, also other host databases available (mouse etc.).	Not specified for human. Ribosome is removed using BLAST against rDNA db.	Not specified.	Not subtracted but reported as part of detection.	Yes, reports identification of human host reads and bacterial mappings.
Means of virus identification	(a) BLAST against a pan-viral database. (b) Remapping of original reads to the identified candidates.	Mapping to the members of the virus database using *bowtie2* [24].	BLAST search against the NCBI nt database.	Protein BLASTP upon two databases. Several tiers of classification of the ORFs.	Not specified.	Taxonomer Binner DB with 21 bp kmers unique identifiers to known viruses.	Custom similarity workflow with hamming distance.
Virus database for identification	A custom database containing 20759 human, animal, plant and bacterial viruses.	Eukaryotic viruses only. Four custom databases available for download.	Specificity is maintained by the subtraction steps prior to assembly and BLAST search.	UniRef 100 peptide database, five annotated protein databases, MetaGenomes On-line.	GAAS tool (https://sourceforge.net/projects/gaas/).	Binner DB needs to be built using KAnalyze [42] (https://sourceforge.net/projects/kanalyze/files/).	TANGO [43] and NCBI Taxonomy [44].
Action when a read maps to different viruses	Score is split among the hit reference sequences.	Not specified.	Not specified.	Not specified.	Not specified.	Assigns as ambiguous.	Parsed for human endogenous retrovirus otherwise classify as ambiguous and discarded.

Most tools use BLAST [23] for initial detection of known references. Vipie uniquely allows web parallel analysis of multi-samples and accounts read hits to multiple viral references for comprehensive population profiling

We aimed to open the possibility of creating a table of viral quantities of multiple samples assessed in parallel by exactly identical processes. Here we introduce Vipie, a web based viral diversity population tool accepting as input a set of files from virome metagenomics NGS analyses of multiple samples. Here we present the workflow and results using NGS samples from Human Microbiome Project and other metagenomics studies. Functional on all modern browsers, the high performance pipeline is freely available for academic usage.

## Implementation

Our pipeline processes de-multiplexed paired FASTQ files, the most typical product of metagenomics sequencing. Several steps are then performed in parallel for all samples: quality control (QC), de-novo assembly of putative genomic contigs, taxonomic classification of the assembled contigs and orphan singleton reads by performing Blast queries against a local custom virus database derived from Genbank, and finally remapping of the sequencing reads onto reference sequences identified by this taxonomic classification. Default analysis parameters can be easily modified (e.g. the QC stringency, or the de novo assembly algorithm).

**Fig. 1:**
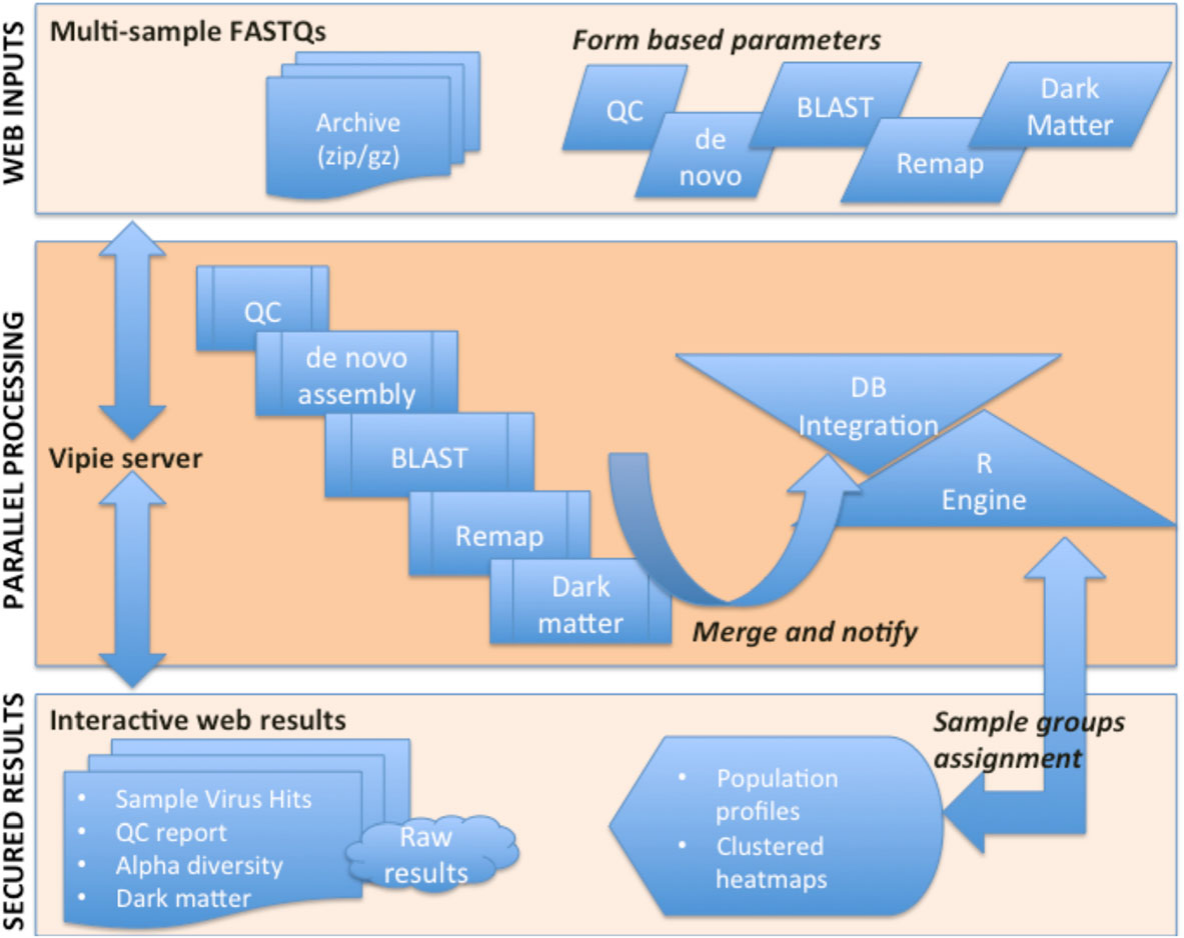
Vipie web flow chart. For efficiency, sample based paired FASTQ files are uploaded as a zipped archive with optional mapping file. Illumina BaseSpace archive downloads can be used without changes. All pipeline parameters can be entered using the web form. The default values and use case are listed in the user guide available at home page along with example multi-sample archive input

Depicted in Fig. 1, Vipie pipeline uses multi processor architecture with integration of PostgreSQL for performance and data management while providing secured interactive results and allowing web form parameters for QC, assembly and scoring. The individual parameters and its default values are listed in the user guide. Trimming and quality control are parameter based applying Galaxy project utilities [13, 14]. We have integrated leading de-novo assembly tools - Velvet [15], MetaVelvet [16], IDBA [17] and MEGAHIT (SOAPDENOVO) [18] and ABySS [19]; these methods and tools are further described and reviewed [5, 20–22]. Taxonomic identification is performed using BLAST [23] against a local NCBI database restricted to whole virus genomes. The final step of the parallel analysis remaps the raw reads using BWA [24] onto a list of best matches from the BLAST queries, and lists the count of original reads matching to each of these references. In cases where reads match equally well to multiple viruses, the score is divided among such best matches to express importantly the ambiguity in assignation of the motifs shared among viral taxa, and the uncertainty of the presently available classification.

De-novo contigs and reads that do not match to any currently known virus, optionally filtered for human genome and known ribosomal DNA, can be retrieved for further analysis as this ‘dark matter’ of the virome presumably containing novel viruses. Our pipeline allows a direct export of these unmapped reads owing to three-step filtering strategy. Reads unmatched to known viruses are first deprived of sequences that match to ribosomal DNA of bacterial, archeal and fungal origin. This is performed by remapping the reads by the BWA program to databases of 16S, 23S and 5S rDNA (a copy of ftp.ncbi.nlm.nih.gov/genomes/TARGET, and a reduced database of 5S rDNA http://www.combio.pl/rrna/) [25]. The next step remaps the reduced set of reads to the human genome. This step yields the potential dark matter of the human genome, mixed with a small proportion of bacterial genomic DNA. Our pipeline does not filter out these bacterial genomic reads, as they may contain novel lysogenic (dormant) phages.

VIPIE’s reference virus database was built from three sources and clustering the sequences to the 97% level of identity further reduced the complexity. First, all viruses were downloaded from the *refseq* database at the NCBI (https://ftp.ncbi.nih.gov/refseq/release/viral/), and reduced to 97% identity by using the CD-HIT program (https://github.com/weizhongli/cdhit/[26]). Then, all virus sequences labeled as “complete”, with the “txid10239” (superkingdom Viruses) in the “Orgn” field were retrieved from Genbank. The query retrieved approximately 80,000 sequences from the database, which were subsequently reduced to the 97% similarity by using the CD-HIT program. Finally, similarly to previous two databases, phages were merged and clustered from the European Bioinformatics Institute (EBI) repository (ftp.ebi.ac.uk/pub/databases/fastafiles/embl_genomes/genomes/Phage/).

The web form, interface dialogs and results are programmed to HTML5 standards and using JavaScript and modern, open source JavaScript libraries (https://jquery.org, https://datatables.net) for browser compatibility. Biopython [27] is used for sequencing parsing and formatting. Parallel processing is achieved via python (https://www.python.org) subprocess module implementation and uses PostgreSQL (https://www.postgresql.org) schema for job tracking and results merging. Standard SMTP library is used for notification, hence the email registration requirement. Clustered heatmaps are implemented with R ggplot2 [28] while other summary and alpha diversity statistics are computed using custom python scripts. Population maps and read distribution count summary charts are created using highcharts.js (https://www.highcharts.com) and custom event handlers for interactivity. Vipie is an ongoing open sourced project and available at https://sourceforge.net/projects/vipie.

## Results

### Input samples and interactive results

The pipeline utility is here demonstrated on set of 11 samples where the input and results are available to all users. The sample set consists of (a) blood, nasal, stool and vagina data from Human Metagenome Project (HMP), (b) diarrhea sample from gastroenteritis outbreak (DRA004165 DNA Data Bank Japan [29, 30]) used in VirusTAP and (c) stool data from in-house ongoing African metagenomics project [31, 32]. Table 2 lists relevant accession identifiers, sources and number of reads along with result links. As the compressed archived exceeds 1.2 gigabytes, a smaller subsampled archive consisting of 20% is available for download on the homepage and the original compressed FASTQ archived is available on https://sourceforge.net/projects/vipie/files/data [33]. End-to-end processing of the 11 samples took 82 min, processing 29,778,980 reads that includes assembly, scoring, and clustering and removal of human reference and known ribosomal references. The performance time was measured after the archive was uploaded as file upload depends fully on local network speed. The interactive results, with population profile maps and filterable viral hit tables are accessible at: https://binf.uta.fi/vipie/results.html?key=eLZPuObVoU. Result links are accessible without registration and designed to be shared among collaborators whereas job history and active jobs are visible only to registered investigators. The results are divided into panels of Population profile & group assignment, QC & Dark matter report, Summary & alpha diversity, and Viral hits table. Raw results, including unmapped dark matter reads that to not match to any known virus can be also downloaded.

**Table 2:** NGS samples used in Vipie validation from Human Microbiome Project, Africa study, and diarrhea sample sourced in Japan gastroenteritis outbreak. ViromeScan listed 20 HMP samples but only Stool types of 4 samples passed QC

AccessionId	Source	Sample Type	Number of Reads^a^	Sample used in Vipie-ViromeScan-VirusTAP validation	Vipie Results^b^
SRS072276	HMP	Blood	438,879	Yes-No-No	1,2
SRS072318	HMP	Blood	753,994	Yes-No-No	1,2
SRS019033	HMP	Retroauricular	1,285,003	Yes-No-No	1
SRS016944	HMP	Retroauricular	1,619,439	Yes-No-No	1
SRS012902	HMP	Stool	2,039,473	Yes-Yes-No	1
SRS014923	HMP	Stool	2,009,179	Yes-Yes-No	1
SRS014466	HMP	Vagina	367,077	Yes-No-No	1,2
SRS015072	HMP	Vagina	495,256	Yes-No-No	1,2
SRS072313	HMP	Nasal	320,672	Yes-No-No	2
SRS072261	HMP	Nasal	367,384	Yes-No-No	2
SRS072366	HMP	Nasal	114,414	Yes-No-No	2
S11	Africa	Stool	1,634,821	Yes-No-No	2
S12	Africa	Stool	1,191,427	Yes-No-No	2
S14	Africa	Stool	1,143,784	Yes-No-No	2
DRA004165	Japan	Diarrheal	1,108,688	Yes-No-Yes	2

In addition to those stool samples, Vipie test archive includes 4 other HMP sample types. Result links with performance time are also provided

^a^Input archive of Result 2 samples (subsampled 20% 225 MB) available at: https://binf.uta.fi/vipie/data/vipie_archive_ssampled.zip

^b^Results 1: https://binf.uta.fi/vipie/results.html?key=2HSPXukkDS (66 min)

Results 2: https://binf.uta.fi/vipie/results.html?key=eLZPuObVoU (82 min)

**Fig. 2:**
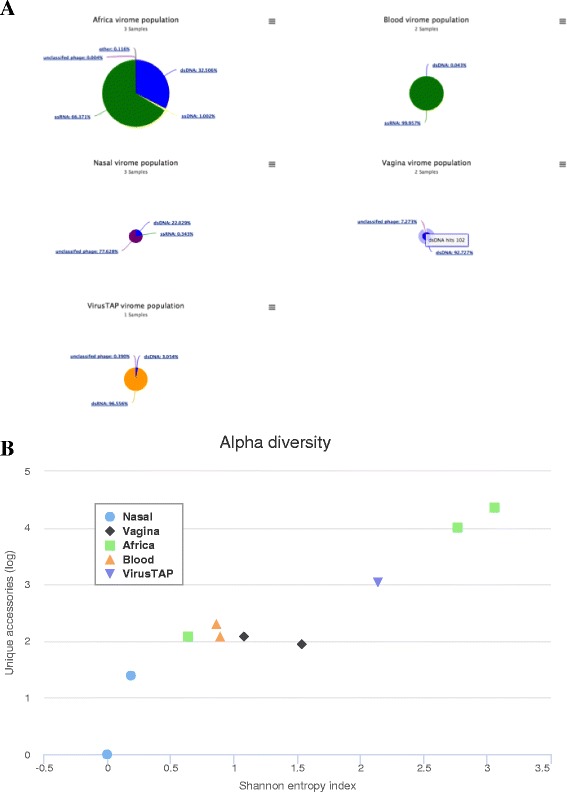
Interactive population profile maps and diversity. Vipie results are securely accessed and browser based. **a** Population chart slices are clickable and their sizes represent relative percentage of relevant taxonomy level. Diarrheal sample is dominated by dsRNA (*orange*) Rotavirus while African stool samples contain ssRNA (*green*) and dsDNA viruses. **b** Alpha diversity is calculated using Shannon entropy. Vipie charts are interactive and can be saved as multiple image formats

Figure 2 shows group-based population pie charts and alpha diversity as measured by Shannon entropy [34]. The population pie chart sizes are relative to total number of hits and their slices are fully interactive as clicking on the slices traverses the taxonomy levels. The tool found 167 unique accessions across the samples and an easy to use searchable and sortable sample hits table is provided and best experienced from the browser, where the table can be collapsed based on taxonomy and sample viral hits can be downloaded as a text file ready for Excel import.

**Fig. 3:**
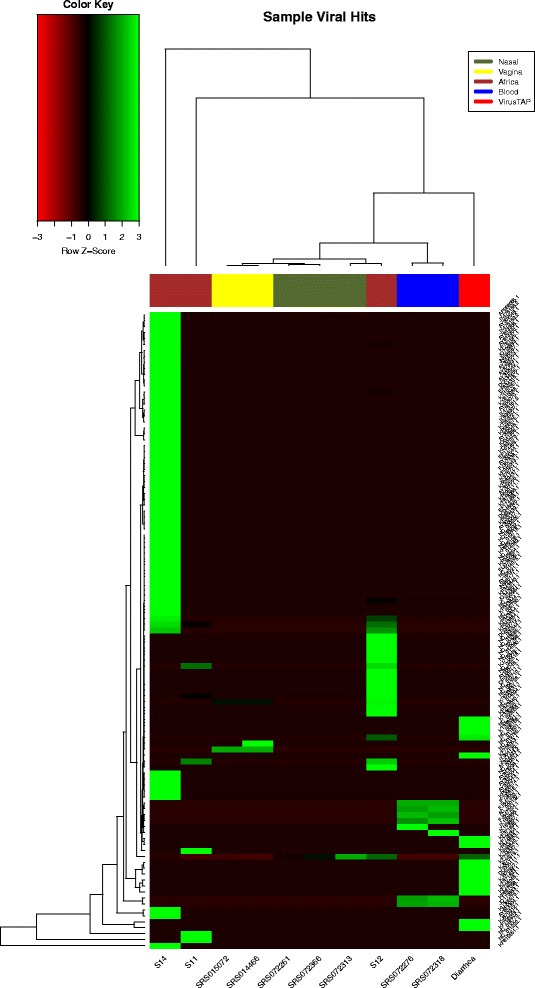
Clustered heatmap of HMP, African and Japanese diarrheal samples. Public NGS data from different consortiums provide opportunities for advanced comparative virome analysis. Healthy HMP sample types clustered correctly (nasal, vaginal, blood samples) while a Japanese sample (gastroenteritis dataset from the VirusTAP report) and African samples (known to be positive for multiple viruses) showed different signatures. HMP samples can be identified using the legend on upper right, with *olive green* for nasal, *yellow* for vagina and *blue* for blood. Samples from rural Africa and VirusTAP (Japan) are marked in *colors brick* and *red*

Our user guide provides screenshots and directions on filtering the sample hits table and using the filtering function, we found Human Herpes hits on a HMP blood sample SRS072276, where herpes in hematological samples have been reported in a prior microbiome and hematopoiesis report [35]. Our results showed that virus population profiles are unique across body sites, reported also in ViromeScan and visually shown in the clustered maps. Interestingly, in the stool sample SRS012902, crAssphage [36] was by far the highest virus detected. Figure 3 shows the clustered heatmap generated in R, and it correctly clustered healthy HMP sample types together [11] while Japanese gastroenteritis and African samples showed profoundly different signatures.

### Comparisons

**Fig. 4:**
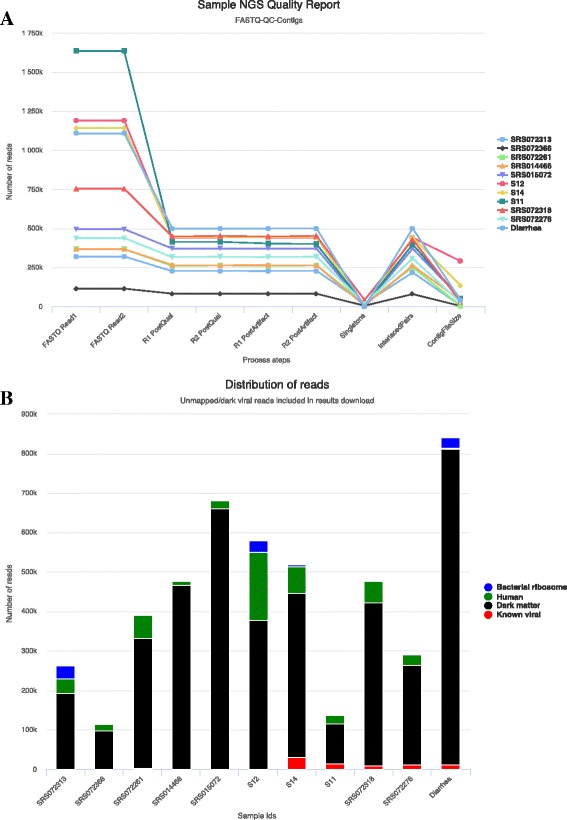
QC and distribution of reads including dark viral matter. **a** The chart shows the number of NGS reads retained per sample through QC, interlacing and de novo assembly. **b** Sample reads, along the x-axis and their aligned origins are shown as stacked bars. Shown in *black*, unmapped viral ‘dark matter’ is of high interest across virology studies. *Blue* bars represent bacterial ribosome, *green* for human while *red* is for known viral matches

We first compared our performance to that of ViromeScan. While ViromeScan states that it supports multiple samples, it requires local installation with 50+ gigabytes of database requirements. The 20 HMP samples used for its validation, only the stool samples passed QC [37] and likely due to timing, the other sample types were not available on HMP download page. Our summary and cluster findings of stool samples and retroauricular, with the highest diversity, samples agree with ViromeScan and other HMP findings of ~5.5 genera per sample [38]. We were unable to reproduce the herpes associations reported with vagina samples as those samples are no longer available. Input parameters, interactive maps, QC report (Fig. 4a) and viral hits of the 11 samples are accessible at https://binf.uta.fi/vipie/results.html?key=eLZPuObVoU and Table 2 contains accession ids along with sample read sizes.

Then performance of Vipie was compared to VirusTAP. Its web based de novo assembly dedicated pipeline required 17 min to process the DRA004165 sample from a study of gastroenteritis [29] in Japan. VirusTAP capably detected 11 Human rotaviruses where this result is cited and also available as its example results. Vipie using the same input detected similar findings of 14 Human rotaviruses strains (shown in Additional file 1: User guide Figure 10B) and also interestingly *Streptococcus* phage strains. Using the same sample, our pipeline required 32 min due to post assembly remapping with custom scoring and then unmapped origin filtering. Because of Vipie’s parallel computing design, the archive of 11 samples and more than 10 times the amount of reads, took just 82 min. The more comprehensive findings also highlight the scoring split strategy on read hits on multiple viruses and investigation of unmapped viral read origins shown in Fig. 4b.

Furthermore, benchmarking was assessed and compared with the recently published MetaShot, using its simulated artificial dataset with a very high share of human sequences mixed with low amounts of many different viral sequences. Table 3 below shows the similar precision and recall results of the two tools. Vipie has a slightly higher percentage of unclassified viral reads likely due to subsampling of the initial dataset, and due to the fact that we optimized the virus BLAST database by removing sequences that were less distant than 3% from its closest relative; similar reduction of taxonomic complexity is known from e.g. bacteriome profiling. The script and Vipie results used for computing this statistics are available with README in Vipie project page on SourceForge. We are grateful to MetaShot authors for permission to use their simulated data, constructed using ART [39].

**Table 3:** (A) Read assignment benchmark assessment of MetaShot and Vipie on simulated dataset^a^ consisting of 19 582 500 human (94.5%), 986 114 bacterial (4.8%) and 146 886 viral (0.7%) reads. Vipie percentages are based on random subsampling of 1 000 000 reads and bacterial statistics are not reported as Vipie reports information on bacterial ribosome only (the bacterial genomic DNA is not filtered out, as it might lead to loss of dormant phage sequences). (B) Precision, Recall and F-measure are calculated on the same data. Input reads and assessment script are available on SourceForge^b^

A	Assigned %^c^		Correctly Assigned %^d^	
	MetaShot	Vipie	MetaShot	Vipie
Human (host)	99.18	99.27	99.99	99.27
Viruses				
Family	97.74	99.98	98.53	93.39
Genus	97.39	98.99	99.75	93.33
Species	97.81	93.66	96.70	92.97
B	Human (host)		Virus	
	MetaShot	Vipie	MetaShot	Vipie
Precision (%)	100.00	100.00	98.30	96.85
Recall (%)	99.97	99.96	98.19	95.36
F-measure (%)	100.00	99.98	98.07	96.08
Unclassified (%)	1.04	0.73	3.94	6.73

^a^ https://recascloud.ba.infn.it/index.php/s/nw4s9hqnF8QkBsK

^b^ https://sourceforge.net/projects/vipie/files/validation/k

^c^The percentage refers to the total number of reads assignable to the specific taxonomic rank

^d^The percentage refers to the relevant assigned reads

### Discussion

Vipie interface is implemented with HTML5 standards and utilizes open source JavaScript libraries. Unlike older and Adobe Flash based applications, Vipie does not require additional installations and supports all modern HTML5 compliant browsers while offering a consistent user experience. The input parameter form is designed to be clean and to group into processed components where each element has custom validation rules. The component details and rules are listed in the user guide. Secured and interactive analysis results are accessed with encrypted links and to promote collaboration, can be shared without registration. Sample based alpha diversity is provided, using Shannon entropy index [34] (Fig. 2) as a representative of diversity methods [35]. Vipie intuitively offers web based, form or file upload sample group reassignment where population and clustered maps are reanalyzed and dynamically redrawn. The pipeline produces a cross tabulation similar to the operational taxonomic unit (OTU) tables from bacteriome profiling, additional statistics is doable with advance R packages such as phyloseq [40] and deseq2 [41].

Often, published pipelines emphasize that their performance is by orders of magnitude faster than existing strategies [7, 8] and that the tasks can be completed in the order of minutes to single hours in a situation where existing viruses account only for a minor fraction of the total read count. We believe that the present Vipie pipeline offers fast data processing for most relevant applications, including real-time assessment of viral repertoire in clinical samples. For comparison, VirusTAP processing, up to assembly with 1 sample (~2 million reads, 172 MBs) took 17 min (Input upload time is not included as it is dependent completely on local network speed.). Vipie process the same sample in 32 min including assembly, cross validation scoring/remapping, known reference filtering and viral dark matter processing. Parallel implementation is ideal for multi-sample processing and input set of 11 samples (Table 2), consisting of ~30 million reads, 1.22 GBs compressed and processed in 82 min. There is no concurrent limit on the number of samples eligible for processing other than a small database overhead. Job completion time has a direct relationship to the sample with the highest read depth and it is well known that interlacing and assembly are high memory tasks. The de novo assembly step implements random subsampling on user defined read percentage, default of 75% with a maximum of 1,000,000 NGS reads per sample. Very large archives can suffer from network timeouts on file upload. In overcoming this scenario, we have successfully deployed Vipie on cluster computing environment and analyze thousands of samples consisting of terabytes of data using SLURM, the default utility for Linux high performance computing. We believe that our strategy offers a good balance between bearable algorithm speed on most machines, and availability of multiple sample processing.

Importantly, the pipeline offers a set of files with bacterial, human, and unknown sequences (the “dark matter” of the virome). Dark matter reads are the remaining unmapped reads after filtering for human and bacterial ribosomes. It has been long known that the unknown dark matter is extremely valuable in virome analysis [9] and in focus with the recent discovery of new bacteriophage virus *crAssphage* while its bacterial host still unknown [36]. Many components of this “dark matter” of the virome have been observed across studies, and are likely to represent existing viruses, yet their taxonomy is presently unknown. The lack of taxonomic classification however should not preclude their use as provisional entities, exposures that are testable and quantifiable in epidemiological studies. Figure 4b shows an interactive sample based chart consisting of stacked bars representing the percentage of reads mapped to human, bacterial ribosomes, known viruses and dark matter. It is apparent that these unmapped reads dominated these NGS samples and deeper advanced analyses are necessary. As such, viral dark matter raw reads are part of downloads.

An often-overlooked aspect is the uncertainty in virus identification. The Genbank database contains many similar isolates of almost every relevant virus serotype. This means that most reads or contigs would map to multiple different sequenced virus isolates. In single sample studies this does not pose any problem - the taxonomy is concluded as the highest scoring hit, or the first of a set of similarly high scoring organisms. This however cannot be done when a pipeline processes multiple samples at the same time: due to the known intrinsic variability of the viruses, even a single subject may produce two different samples where different virus quasi-species may prevail that will preferentially map to two different virus reference sequences. There are two possible solutions to the problem: the ViromeScan pipeline employed one where the databases are smaller with a limited scope. Unfortunately, the strategy towards their construction was not described in the paper, but clearly only the most important serotypes represent each virus species - e.g. only 92 sequences cover the whole repertoire of human DNA viruses. In Vipie we chose a different strategy: we decided to build a representative virus database of all available sequences (clustered to a 97% similarity level for the sake of algorithm speed), and all multiple equally likely mapping hits are resolved by splitting the mapping score among the different hits. At higher taxonomic levels of family or genus this is not visible, but when descending to the level below species (to individual reference sequences), the uncertainty is expressed by the existence of a whole block of candidate viral reference sequences to which the sample distributes many of its reads. This should express that the found virus is similar to many references, but neither is fully identical. This strategy has proven feasible in our benchmarking experiment when we reached parameters reasonably close to the specialized single-sample taxonomy tool MetaShot [12], while offering the possibility of parallel assessment of multiple viromes in one run. We assigned 3.73% less reads to their correct species (MetaShot 96.70%, VIPIE 92.97%) - this may be (a) the effect of clustering our representative virus database; some reads falling into species or serotype specific viral regions may thus remain unidentified; (b) the consequence of subsampling - VIPIE uses subsampling to 1 million reads maximum, whereas the simulated MetaShot data set is more than 20 times larger, with most of the viruses in trace amounts.

## Conclusions

Virome NGS datasets are unique in several aspects. Firstly, unlike in amplicon libraries in bacteriome profiling, there are no clearly outlined methods of taxonomic classification and of quantification of the viral agents. Secondly, unlike work on e.g. RNA sequencing in humans and animals, there is no well-defined reference set of viral sequences. Therefore the virome characterization must rely on an insufficient knowledge of existing viruses, and on still uncertain techniques of taxonomic sorting - first because the taxonomy of viruses is still rapidly evolving.

When studying an association of existing or novel viral agents with a condition (as is a disease, an ecological variable, or a human intervention), it is imperative to keep the analytical conditions identical across the data set, and to attempt a truly unbiased relative quantification of the viral agents present therein. This can be safely achieved only if all samples of the dataset are processed by an identical protocol - and if they are quantified against a common set of reference sequences. The reference set should be a union of all possible references of the whole study set. Our pipeline performs such quantification: it identifies all agents present in the dataset and in the final step it attempts remapping of the original reads from every sample to this whole reference set. This enables employing the ensuing virus quantity tables in downstream analyses similarly to the well-established analyses of bacterial profiles from 16S rDNA mass sequencing.

## Availability and requirements

**Project name:** Vipie: web pipeline for parallel characterization of viral population from multiple NGS samples

**Project home page:** https://binf.uta.fi/vipie

**Source code:** https://sourceforge.net/projects/vipie

**Operating system(s):** Platform independent

**Programming language:** Python 2.7+, R 3.3, JavaScript, HTML5, PostgreSQL 9+

**License:** Vipie is available free of charge to academic and non-profit institutions.

**Any restrictions to use by non-academics:** Please contact authors for commercial use.

## Additional file

Additional file 1: Vipie User Guide. (DOCX 3189 kb)

## Abbreviations

HMP: Human microbiome project
NGS: Next generation sequencing
OTU: Operational taxonomic unit
QC: Quality control

## References

[1] The Human Microbiome Consortium (2012). Structure, function and diversity of the healthy human microbiome. Nature.

[2] Turnbaugh PJ, Ley RE, Hamady M, Fraser-Liggett CM, Knight R, Gordon JI (2007). The human microbiome project. Nature.

[3] Houldcroft CJ, Beale MA, Breuer J (2017). Clinical and biological insights from viral genome sequencing. Nat Rev Microbiol.

[4] Tringe SG, Rubin EM (2005). Metagenomics: DNA sequencing of environmental samples. Nat Rev Genet.

[5] Shapton TJ (2014). An introduction to the analysis of shotgun metagenomic data. Front Plant Sci.

[6] Flygare S, Simon K (2016). Taxonomer: an interactive metagenomics analysis portal for universal pathogen detection and host mRNA expression profiling. Genome Biol.

[7] Yamashita A (2016). VirusTAP: viral genome-targeted assembly pipeline. Front Microbiol.

[8] Wommack KE, Bhavsar J (2012). VIROME: a standard operating procedure for analysis of viral metagenome sequences. Stand Genomic Sci.

[9] Roux S, Faubladier M (2011). Metavir: a web server dedicated to virome analysis. Bioinformatics.

[10] Roux S (2014). Metavir 2: new tools for viral metagenome comparison and assembled virome analysis. BMC Bioinf.

[11] Rampelli S, Soverini M (2016). ViromeScan: a new tool for metagenomic viral community profiling. BMC Genomics.

[12] Fosso B. et al. MetaShot: an accurate workflow for taxon classification of host-associated microbiome from shotgun metagenomic data. Bioinform. 2017. doi: 10.1093/bioinformatics/btx036. 10.1093/bioinformatics/btx036 PMC5447231 28130230

[13] Afgan E, Taylor J, Anton Nekrutenko A, Goecks J (2016). The galaxy platform for accessible, reproducible and collaborative biomedical analyses: 2016 update. Nucleic Acids Res.

[14] Blankenberg D, Taylor J, Nekrutenko A, the Galaxy Team (2014). Dissemination of scientific software with galaxy ToolShed. Genome Biol.

[15] Zerbina DR, Birney E (2008). Velvet: algorithms for de novo short read assembly using de Bruijn graphs. Genome Res.

[16] Namiki T, Hachiya T, Tanaka H, Sakakibara Y (2012). MetaVelvet : An extension of Velvet assembler to de novo metagenome assembly from short sequence reads. Nucleic Acids Res.

[17] Peng Y (2013). IDBA-UD: a de novo assembler for single-cell and metagenomic sequencing data with highly uneven depth. Bioinformatics.

[18] Li D (2015). MEGAHIT: an ultra-fast single-node solution for large and complex metagenomics assembly via succinct de Bruijn graph. Bioinformatics.

[19] Simpson K (2009). ABySS: A parallel assembler for short read sequence data. Genome Res.

[20] Paszkiewicz K, Studholme DJ (2010). De novo assembly of short sequence reads. Brief Bioinform.

[21] Tritt A, Eisen JA, Facciotti MT, Darling AE (2012). An integrated pipeline for de novo assembly of microbial genomes. PLoS One.

[22] Li Y (2016). VIP: an integrated pipeline for metagenomics of virus identification and discovery. Sci Rep.

[23] Altschul SF (1990). Basic local alignment search tool. J Mol Biol.

[24] Li H, Durbin R (2009). Fast and accurate short read alignment with Burrows-Wheeler transform. Bioinformatics.

[25] Szymanski M, Zielezinski A (2016). 5SRNAdb: an information resource for 5S ribosomal RNAs. Nucleic Acids Res.

[26] Edgar RC (2010). Search and clustering orders of magnitude faster than BLAST. Bioinformatics.

[27] Cock PA, Antao T, Chang JT, Bradman BA, Cox CJ, Dalke A, Friedberg I, Hamelryck T, Kauff F, Wilczynski B, de Hoon MJL (2009). Biopython: freely available python tools for computational molecular biology and bioinformatics. Bioinformatics.

[28] Wickham H (2009). ggplot2: elegant graphics for data analysis.

[29] Kimura H (2014). A food-borne outbreak of gastroenteritis due to genotype G1P[8] rotavirus among adolescents in Japan. Microbiol Immunol.

[30] DNA Data bank of Japan http://getentry.ddbj.nig.ac.jp/(DRA004165) Accessed 01 Dec 2016.

[31] Rodríguez-Diaz J (2014). Presence of human enteric viruses in the stools of healthy Malawian 6-month-old infants. J Pediatr Gastroenterol Nutr.

[32] Mangani C (2015). Effect of complementary feeding with lipid-based nutrient supplements and corn-soy blend on the incidence of stunting and linear growth among 6- to 18-month-old infants and children in rural Malawi. Matern Child Nutr.

[33] Vipie project SourceForge https://sourceforge.net/projects/vipie/files/data/Accessed 15 Mar 2017

[34] Shannon CE (1948). A mathematical theory of communication. Bell Syst Tech J.

[35] Simpson EH (1949). Measurement of diversity. Nature.

[36] Dutilh BE, Edwards RA (2014). A highly abundant bacteriophage discovered in the unknown sequences of human faecal metagenomes. Nat Commun.

[37] NIH Human Microbiome Project website. http://www.hmpdacc.org/HMASM/HMASM-690.csv. Accessed 01 Jan 2017

[38] Wylie KM, Mihindukulasuriya KA, Zhou Y, Sodergren E, Storch GA, Weinstock GM (2014). Metagenomic analysis of double-stranded DNA viruses in healthy adults. BMC Biol.

[39] Huang W (2012). ART: a next-generation sequencing read simulator. Bioinformatics.

[40] McMurdie PJ, Holmes S (2013). Phyloseq: an R package for reproducible interactive analysis and graphics of microbiome census data. PLoS One.

[41] Love MI, Huber W, Anders S (2014). Moderated estimation of fold change and dispersion for RNA-seq data with DESeq2. Genome Biol.

[42] Audano P, Vannberg F (2014). KAnalyze: a fast versatile pipelined k-mer toolkit. Bioinformatics.

[43] Alonso-Alemany D (2014). Further steps in TANGO: improved taxonomic assignment in metagenomics. Bioinformatics.

[44] Sayers EW (2009). Database resources of the national center for biotechnology information. Nucleic Acids Res.

