# Supplementary material for: Vipie: web pipeline for parallel characterization of viral populations from multiple NGS samples
Source: BMC Genomics. 2017 May 15;18:378. doi: 10.1186/s12864-017-3721-7 (PMC5430618; doi:10.1186/s12864-017-3721-7)
Supplement: Additional file 1: — Vipie User Guide. (DOCX 3189 kb) [file 12864_2017_3721_MOESM1_ESM.docx]

**Vipie: web pipeline for parallel characterization of viral populations from multiple NGS samples**

**User manual**

**Current as of March 24^th^, 2017**

**
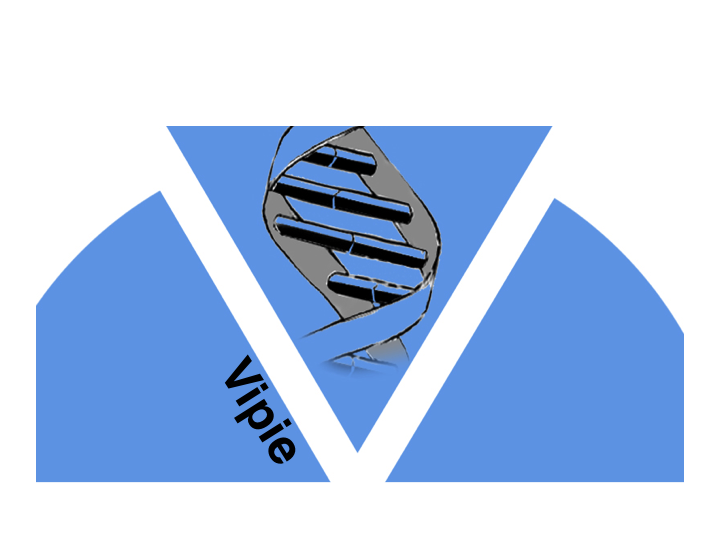
**

Table of Contents

Vipie Overview 3

Workflow 4

Home page 5

Parameters 5

Paired fastq files 7

Sample group mapping file 8

Submission, performance and limitations 8

Blacklisted accessions 9

Vipie Results 9

Results layout 10

Download 10

Diversity Plots 11

Summary Boxplots 12

Sample Viral Matched table 13

Clustered heatmaps 14

The sample viral hit table is essentially a numeric matrix and such data structures can be clustered, where the inter-sample (columns) distances are computed on the viral hit similarities. Figure 10 is downloaded from results. 15

Mapping assignment post analysis 17

QC, Mapping assignment low reads (NA) and Exclude controls 17

Viral ‘dark matter’ and distribution of reads 18

User case application – virome population profile results of 11 samples from Human Microbiome Project (HMP), DNA Bank of Japan and in-house Africa metagenomics project. 20

Sample protocol preparation 20

Parameters 21

Results 21

Custom sample group maps 21

Contact 24

# Vipie Overview

**Vi**rome **Pi**peline **E**xtraction (https://binf.uta.fi/vipie) is a metagenomics analysis project allowing web based viral population and diversity detection from next generation sequencing of **multiple samples**, using parallel processing on the individual sample sequencing files compressed inside an archive (.zip/.gz) file. Figure 1 shows the web architecture and major components. Required and optional parameters, with accepted values, are listed below. Vipie has been tested on all modern browsers. An active and open sourced project, the code is deposited at: https://sourceforge.net/projects/vipie

| 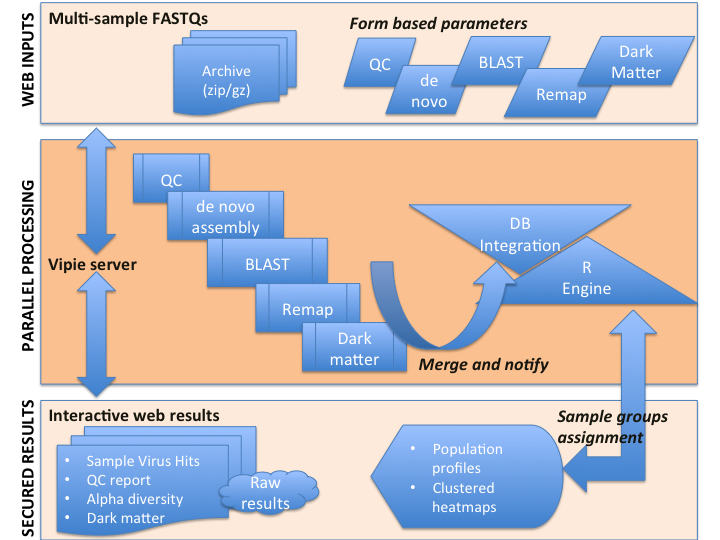 |
| --- |

Figure 1 Vipie allows web based multi-sample processing of viral extractions via compressed archived FASTQ files, such as from Illumina BaseSpace downloads, with sample group assignment support. Parameters are also web-based and more details are listed in the workflow section. In addition, example visualizations are shown and described in the results and user scenario sections.

Usage is free for non-profit academic research. Please see manuscript for in depth background, results and discussions along with citations and references.

The primary functions are:

- Process multi-sample FASTQ archives (zip/gz)
- Web based flexible QC, assembly and mapping parameter settings
- Filtering human and bacterial reads
- Interactive population pie charts, R clustered heatmaps
- QC and distribution of reads report and alpha (Shannon index) diversity plots
- Dynamic sample group reassignment and plotting
- Sortable and downloadable viral matched hits table
- All plots and raw results are downloadable

Singular results can be securely accessed and shared without registration, with encrypted URL to facilitate collaborations. Vipie is optimized for HTML5 compatible browsers and has been extensively tested on recent versions of Chrome, Firefox and Opera. The application is functional on Internet Explorer 11+ but some visualization might not due to incompatible JavaScript libraries.

Below we will go over the workflow including the interface and parameters. After the workflow we will go over the results from the example input provided for testing and available on the main page. This example input archive (172 megabytes), with 13 samples is randomly selected, 40K to 118K reads, from an unpublished and ongoing stool metagenomics project studying virome diversity in children.

This smaller archive is available here:

<https://binf.uta.fi/vipie/data/selected.zip>

While the full archive (1.2 gigabytes) used in the manuscript is available on source forge:

<https://sourceforge.net/projects/vipie/files/data/>

The submission uses entirely default parameters values, listed below and the analysis takes roughly 8 minutes. The results, accessible without registration, are available at <https://binf.uta.fi/vipie/results.html?key=haVZuv070b>

Please note that usage of this dataset is solely for Vipie testing and any other usage or reference requires explicit consent.

The final section of this guide contains an advanced use case that was included as demo results in our manuscript. The 11 samples originate from Human Microbiome Project, DNA Bank of Japan and in house metagenomics projects. The samples have different sampled sources forming natural group assignments and used to highlight population profiles. While figures shown in this guide can be downloaded using the image download function, Vipie results are best accessed on a modern browser to take advantage of tool tips on mouse overs, drilling on clicks and sub searches.

## Workflow

Our pipeline performs virome population analysis with paired reads using de novo assembly, alignment, accession scoring and then remapping. Prior to assembly, quality control and filtering are done with Galaxy utilities. De novo assembly is accomplished via selection of velvet/meta-velvet/IDBA-UD/MEGAHIT, alignment and scoring with local BLAST and subsequent remapping with BWA. Relevant parameters are passed in via the browser, as described in detail in the Parameters section below.

While the web interface is written in HTML5 and JavaScript, the server and bioinformatics integration scripts are written in Python with library bindings including Biopython, Numpy, Scipy and PostresSQL. R, a statistical open sourced language, is used for clustering and plotting. Email messages are used for status notification, including initial submission, errors and completion.

## Home page

The website is organized into job entry interface and then an independent shareable results page. Prior to job submission, user need to register with email, encrypted password and home contact institution. An example is shown in figure 2 below.


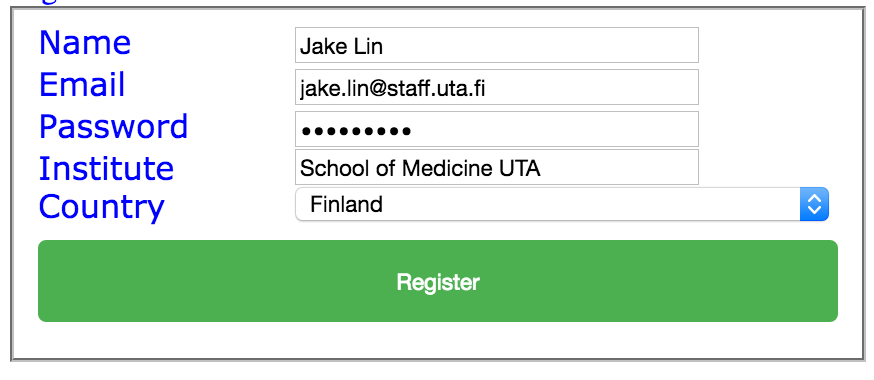


Figure 2 Account registration is needed to securely submit jobs and receive results.

Upon registration and approval, users are free to submit jobs after logging on. The default home interface is shown below, where a project name and upload of FASTQ archive (zip/tar.gz) are required for job submission. Job parameters, such as QC and alignment, can be set dynamically and their default and accepted values are listed in the Parameters section below. Ongoing and completed jobs are listed, shown in Figure 3 below, and results are accessible by clicking on the Completed link. Active (less than 30 days old) jobs can be re-analysis without file upload by clicking the ReRun analysis link.


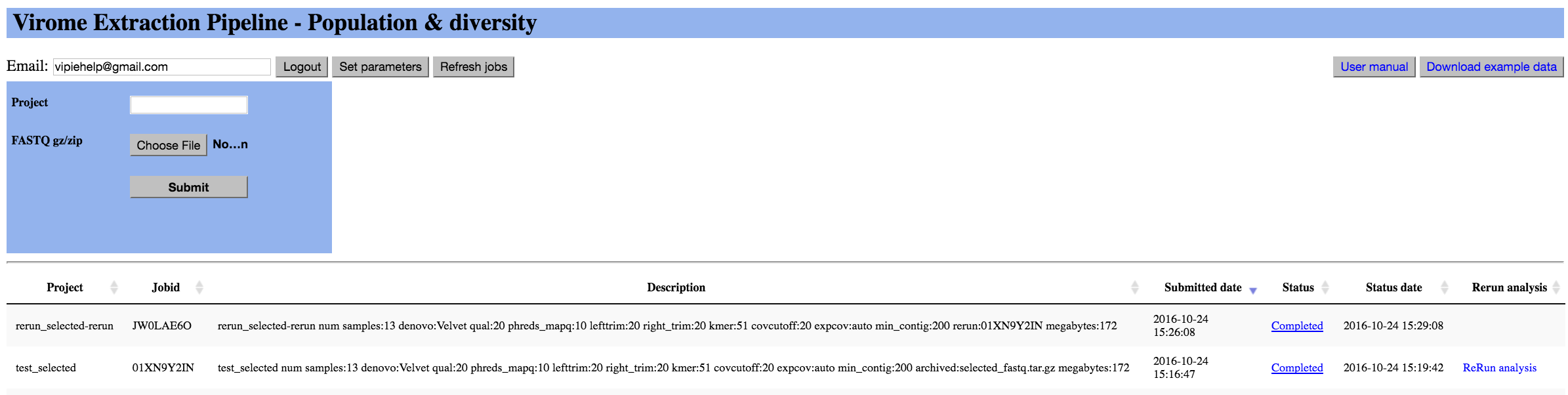


Figure 3 The metagenomics pipeline requires uploading a zip file containing paired fastq files. Ongoing and completed jobs are listed with results and rerun links. Pipeline parameters can be updated and described in the next section.

## Parameters

As stated, the only required input prior to submission is project name and selection of a zip or gz file containing valid paired FASTQ files (R1 and R2 for read 1 and read 2). Using the Set parameters link, the default pipeline parameters can be easily updated. Mouse over of the input fields will show descriptions and valid range of values. These values are strictly enforced and the individual parameters are discussed below. Figure 4 shows the sections of QC, de novo assembly, BLAST and REMAP parameters.

| 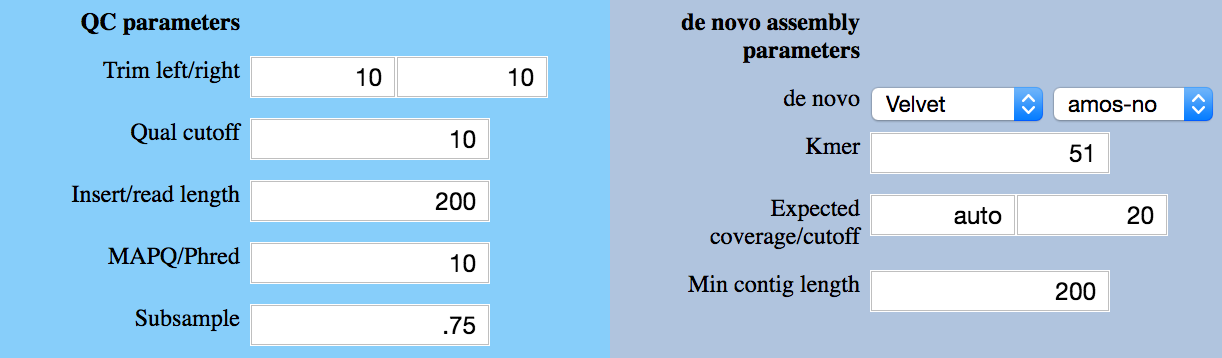 |
| --- |
| 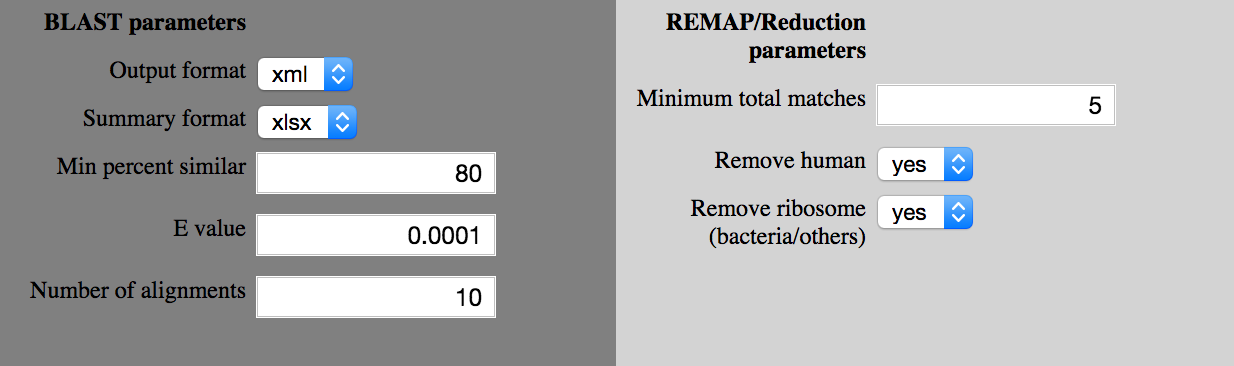 |

Figure 4 Browser adjustable pipeline parameters are form based, such as QC trimming and coverage filters. Human and known ribosome genomic references are also optionally removed after known viral mapping processing.

The order listed reflected beginning from the top left and down, across the columns. The default values are enclosed in parentheses.

**QC**:

1. QC Trim left/right – numeric (20)/(20), trims indicated left and right bases that will be trimmed regardless of their quality. Useful e.g. when a preamplification adaptor is utilized in the wet lab protocol.
2. Qual cutoff – numeric (20), defines a minimum base quality filtering
3. Insert length – numeric (200), defines an estimated read insert length
4. MAPQ Phred overall – numeric (10), metric indicating minimum overall read quality
5. Subsample – numeric (.75 or 75%) [0 < x < 1] – the portion of input reads used for denovo assembly preparation. As most representative reads are repeated and assembly is memory intensive, a subset of the input reads is sufficient and in this case, 75% of the reads, up to a million are randomly selected. Samples with low NGS reads, less than 100,000 are not subsampled.

De novo assembly:

1. De novo assembly algorithm – dropdown (Velvet), Velvet, Metavelvet, IDBA, MEGAHIT and ABySS. The assemblers listed are all published and well cited. IDBA is designed for uneven depth. We encourage readers to look at the publications and websites for more details. From our testing, the final hit/matched results are similar and velvet is the fastest.
2. Amos – (No) produces alignment file viewable e.g. in the Tablet software (https://ics.hutton.ac.uk/tablet/ ). The file can be quite large.
3. Kmer – numeric (51), must be > 21 and odd, indicates word length for graph construction. The longer K gives longer contigs but also less sensitive and computationally cost. The largest K allowed is 101.
4. Expected coverage and coverage cutoff, auto and numeric (auto), (20), used by Velvet along with K to optimize de novo assembly/contig constructions.
5. Min contig length – numeric (100), generally it should be around double of indicated K.

BLAST:

1. Output format – dropdown selection (xml)/(csv), execution can be more sensitive with xml as it captures fragment hits (hsp) though longer processing compared to tsv.
2. Summary format, - dropdown (xlsx) refers to summary raw output and it also supports the smaller text based csv format.
3. E Value – decimal (.0001), used by BLAST for filtering. Please note that the smaller the E Value, the more significant is the matched. See [BLAST FAQ](https://blast.ncbi.nlm.nih.gov/Blast.cgi?CMD=Web&PAGE_TYPE=BlastDocs&DOC_TYPE=FAQ) for more details.
4. Num alignment – integer (10), used by BLAST as minimum depth of alignment. See BLAST FAQ for more details.
5. Min percent similar – numeric (80) 1 .. 100, indicates minimum percentage similarity of contig bases to reference

REMAP:

1. Min acceptable hits – numeric (5), positive integers 1, viral matches will be filtered if the total counts across the samples are less than set value
2. Hits PER – numeric (100,000) – Input reads are remapped to best accessions and the accessions are assigned a proportion fraction. This fraction is multiplied by Hits PER and reported on the sample match table.
3. Apply blacklist – dropdown yes/no – defaults to yes, and blacklisted accessions consisting of vector and synthetic viral references are removed. The list of accessions is listed below on page 9.
4. Remove human – dropdown yes/no – defaults to yes, removes unmapped reads matching HG-37
5. Remove bacterial ribosomes yes/no – defaults to yes, removes unmapped reads matching rDNA (16S, 23S and 5S) from NCBI

## Paired fastq files

The pipeline is primarily written for the Illumina MiSeq platform, which produces paired short reads for metagenomics. This implies that there are two files per sample, *R1 and *R2 and they must be named consistently as they have to be merged.

For example:

S2_L001_R1_001.fastq

S2_L001_R2_001.fastq

DS1_L001_R1_001.fastq.gz

DS1_L001_R2_001.fastq.gz

DS2_L001_R1_001.fastq.gz

DS2_L001_R2_001.fastq.gz

S2, DS1 and DS2 are correct paired samples, with R1 and R2 read files. It is required that you compressed the FASTQ files; here we used Linux gzip tool as Vipie accepts archives with extension gz or zip.

## Sample group mapping file

While creating and uploading the input archive, you can optionally include a mapping.xlsx/txt, as shown in Figure 5 to defined groups for sample sets. Sample group assignments can also be done after the analysis, from results page panel Population profile & group assignment using the form shown in Figure 12.

These sample groups can be any string description and examples group labels are case and controls, time points, or samples from geographical locations. The figured below captured from Excel shows 13 samples from available example input (column A) assigned to 3 groups (column B) of control, case_grp1 and case_grp2. As each category is assigned a unique color and label, maximum of 10 categories is recommended because of crowding and spatial limitations. Users can freely reassign and perform unlimited replots as the original results do not changed. The format is straightforward where each line defines a sample to a group.


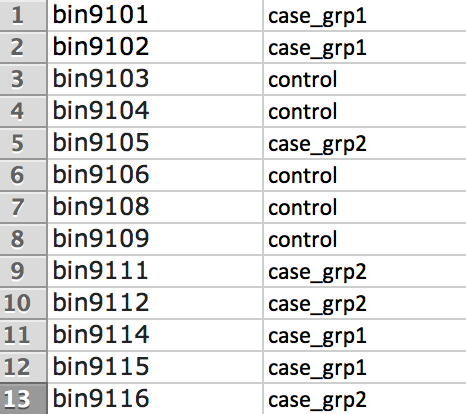


Figure 5 Sample names included in the FASTQ files can be assigned to different groups for clustering and population profiling plots. Assignments included in mapping.txt in archive input are automatically processed and the association can be assigned manually in the default Population profile of the results panel.

## Submission, performance and limitations

For usability and performance, there is a maximum upper bound file size of 20 gigabytes for the archived compressed files. This translates to approximately 100 gigabytes uncompressed FASTQ reads. We encourage researchers with larger files to contact us directly for offline execution. Local installation of Vipie is possible as source code is available on SourceForge (<https://sourceforge.net/projects/vipie/>). The architecture diagram from figure 1 lists language and component dependencies. From our testing, large archives can encounter fatal network timeouts, which is completely dependent on network provider. Vipie is programmed with multi processing where each sample is processed independently; meaning the submission is not completed until every sample is processed. Please note that web file uploads have considerable dependence on network speed. It is recommended to use the application only with high-speed networks. Currently, there is a maximum of 10 active jobs per user and this limit might be adjusted dependent on traffic and resource availability.

## Blacklisted accessions

Vector and synthetic viral accessions are recommended (optionally) removed from population profiling. The current accessions are listed here and will be updated as necessary.

**Table 1**. Vector and synthetic accessions are optionally blacklisted to improve accuracy.

| AF324493.2 | **HIV-1 vector pNL4-3, complete sequence**. |
| --- | --- |
| AY656167.1 | Chimeric dengue virus vector p4-D3L-ME, complete sequence. |
| AY656169.1 | Dengue virus type 3 vector p3, complete sequence. |
| AY705791.1 | Borna disease virus rescue plasmid pBRT7-HrBDVc, complete sequence. |
| AY744148.1 | Dengue virus type 2 vector p2, complete sequence. |
| FJ436096.1 | Synthetic construct Gallid herpesvirus 2 clone pC12/130-10, complete sequence. |
| FJ436097.1 | Synthetic construct Gallid herpesvirus 2 clone pC12/130-15, complete sequence. |
| FJ593289.1 | Human herpesvirus 1 transgenic strain 17, complete genome. |
| GU179001.1 | Human herpesvirus 5 transgenic strain Merlin, complete genome. |
| GU474419.1 | Synthetic construct modified HIV-1 subtype C backbone, complete sequence. |
| GU980198.1 | Human herpesvirus 5 transgenic strain CINCY+Towne, complete genome. |
| HQ687214.1 | Virus-induced gene silencing vector pCAPE2-PsPDS, complete sequence. |
| KF022001.1 | Autographa californica nucleopolyhedrovirus transgenic, complete sequence. |
| KF493877.1 | Human herpesvirus 5 transgenic isolate Towne-BAC-der, complete genome. |
| KJ540270.1 | Vibrio phage CTX plasmid pCTX-3 Kan, complete sequence. |
| KP343683.1 | **Cyprinid herpesvirus 3 isolate FL BAC revertant ORF136 Luc, complete genome**. |
| KR093640.1 | Moraxella phage Mcat16, complete genome. |
| AY376438.1 | Dengue virus vector p4(Delta30) |
| KX576684.1 | Zika virus vector pZIKV-ICD, complete sequence. |

# Vipie Results

Submissions can take a few minutes to hours dependent on the number of samples, depth of coverage of samples and also on the pipeline parameters. File upload is fully dependent on network speed. Upon submission and upload, a job-started email is sent and this email contains a secured URL to check processing status. Upon job completion, meaning all samples processed (completed or error), an email is sent with a link to plots and downloadable results. Inputs and outputs are kept confidential and will be kept in our secured server for 30 days. To improve usability, original submissions can be rerun. Web plots are freely downloadable and dynamically updated on group reassignment actions.

## Results layout


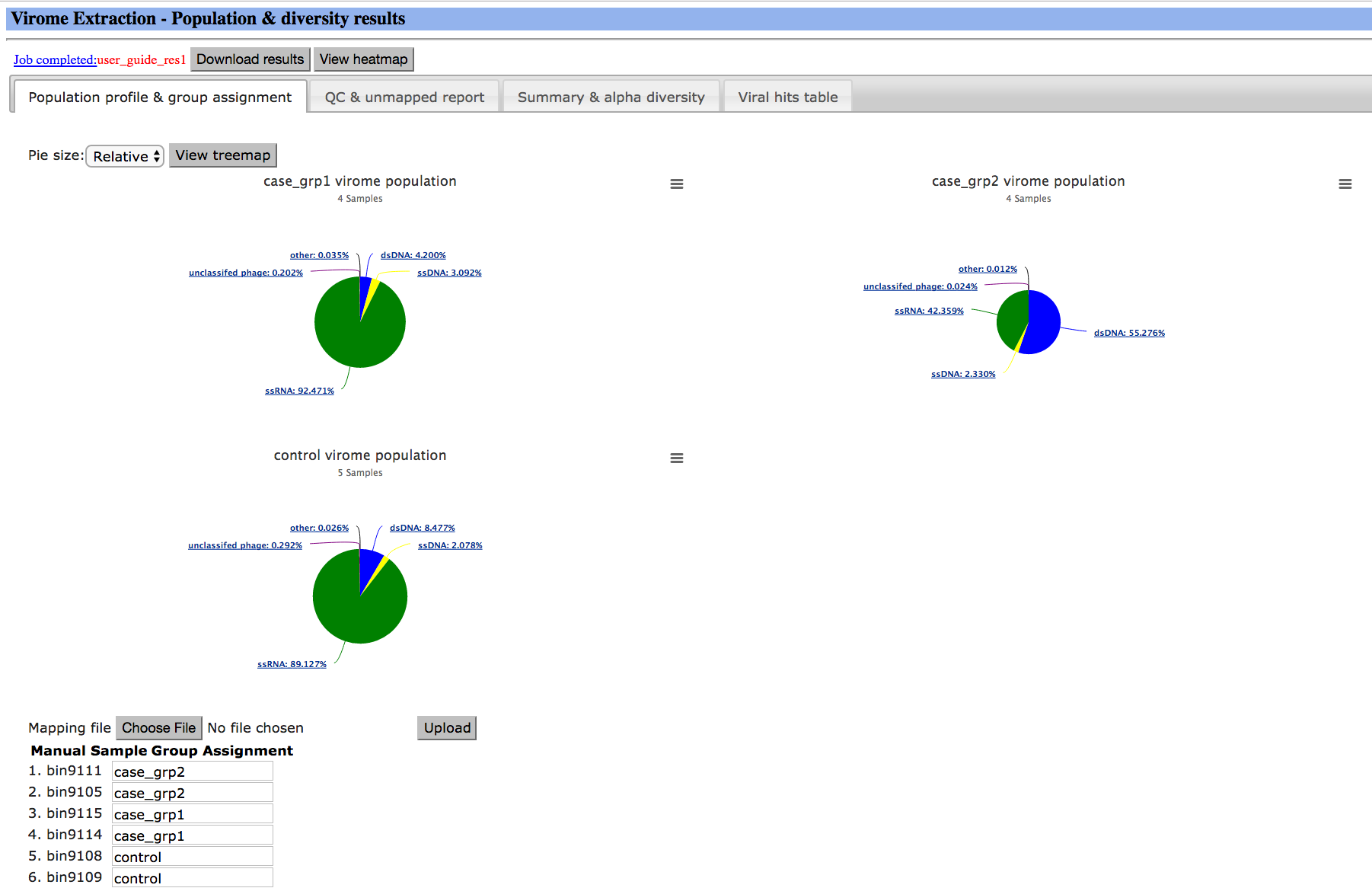


Figure 6 Results are divided into 4 panels, with Population profile & group assignment, QC & unmapped report, Summary & alpha diversity and then the sortable and searchable Viral hits table. Population maps are drillable to accessory identification and their sizes can be viewed with relative total hits ratio or uniform across the groups. Raw results are available as a download for up to 30 days.

All the Result section figures shown are obtained from the provided example file (13 samples, 40,000 to 118,000 reads), using default parameters and accessible here:

<https://binf.uta.fi/vipie/results.html?key=haVZuv070b>

Figure 6 is the default results page accessible on web browsers. The raw results are downloadable as zipped file and the content is described in Figure 7. Results are calculated to show taxonomy level proportion sizes and are organized by optionally assigned groups. Interactive population pie charts are shown in Figure 8. Unassigned samples can be re-plotted from the web results and discussed in the Mapping assignment Post analysis section below.

## Download

The raw tables, contigs, unmatched contigs, summary output and clustered heatmap/dendrogram from R are downloaded as [JobKey].tar.gz after clicking on Download results. The file can be unzipped using most zip software, including gunzip, winzip and 7zip. Below is a snapshot after inflating the tar.gz file.


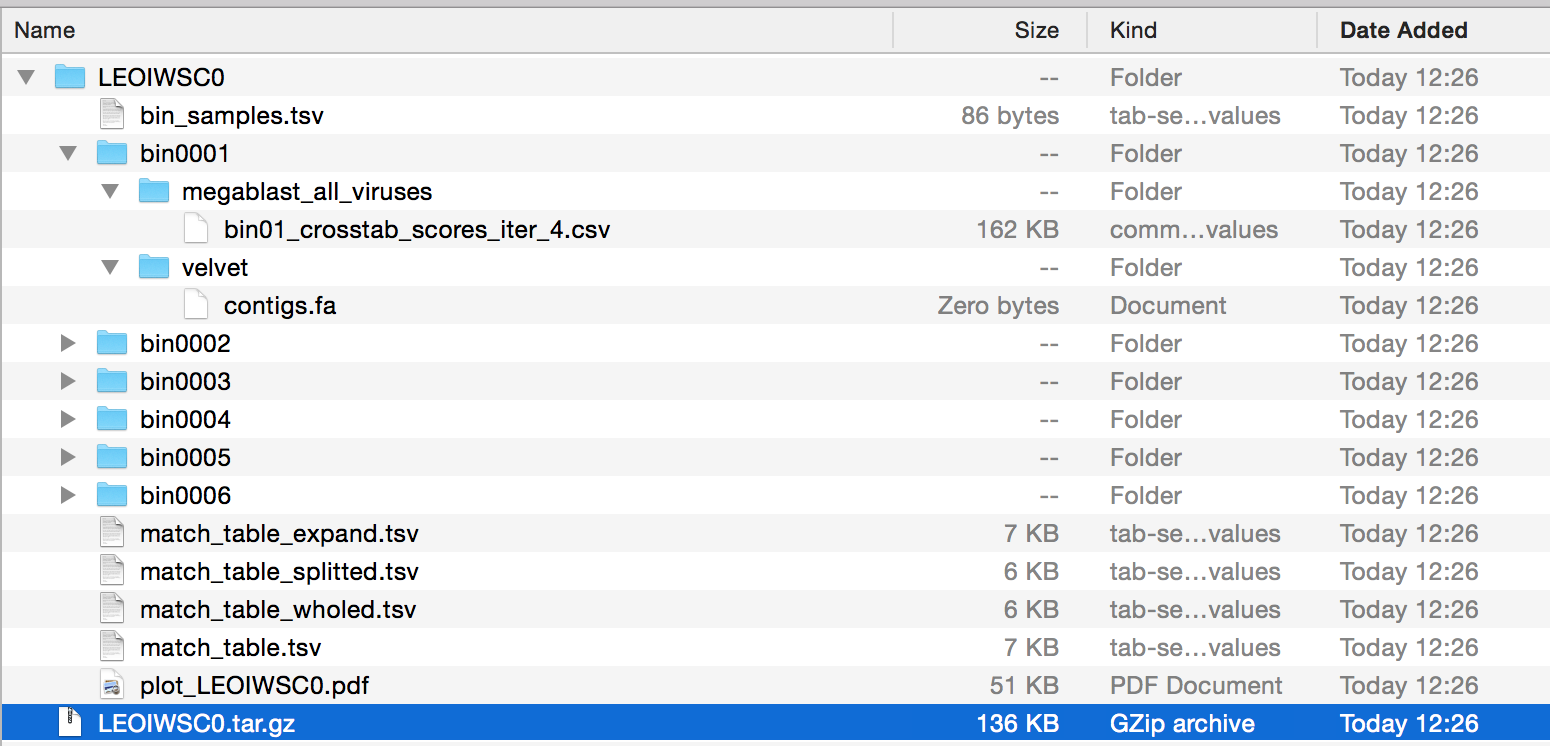


Figure 7 Vipie results download. The unzipped download consists of merged text sample virus tables and assembled contigs and mapped reads organized in processing bins. Sample name to bin number is shown in bin_samples.tsv as well as on web results.

Sequencing data are stored in bins (usually correspond to one combination of indices in the sequencing run), and the relationship between bins and their samples is stored in bin_samples.tsv, as seen in figure 7.

For each bin, the contigs from de novo assembly are provided. At the bin level we also provide BLAST summary results with pair similarity scores. In addition, the match tables and plot are provided.

Obviously, if a read or contig maps to a genomic segment shared by many related viruses, it is impossible to disentangle which of the viruses is really present in the sample. An example may be a hit to a region shared by many virus serotypes. Vipie solves it by splitting the hit score among all equally probable hits. Consequently, the list of identified viruses contains all possible hits, but only those with the highest score are probably present in the sample.

## Diversity Plots

**A**


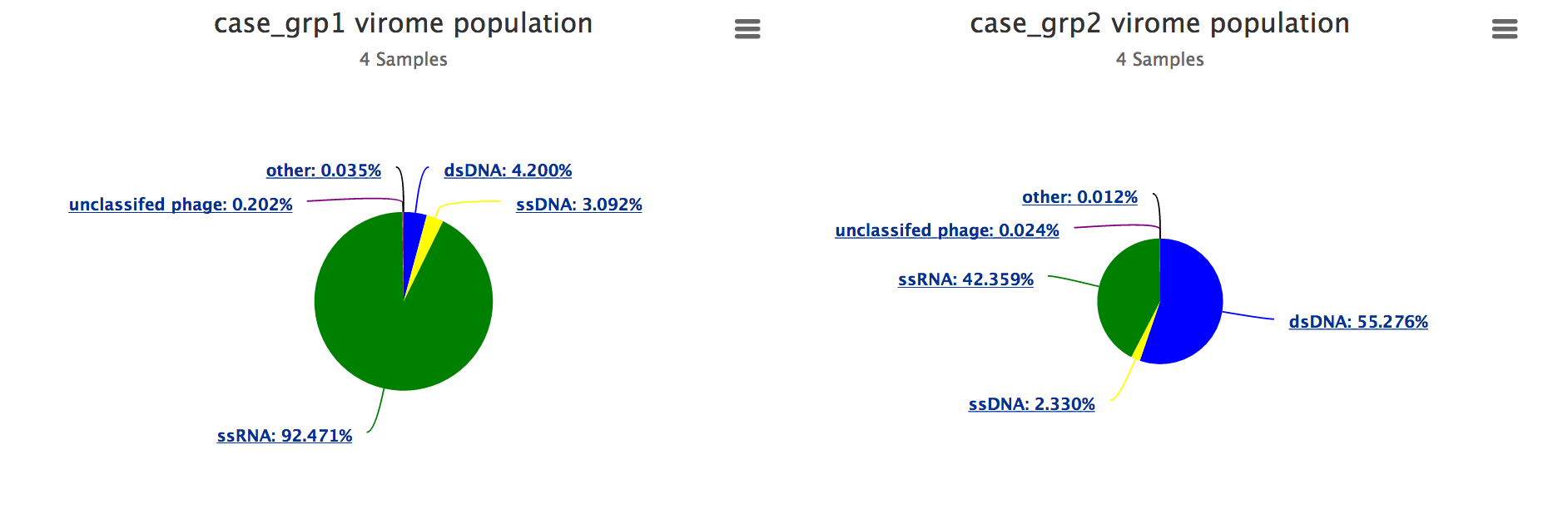


**B**
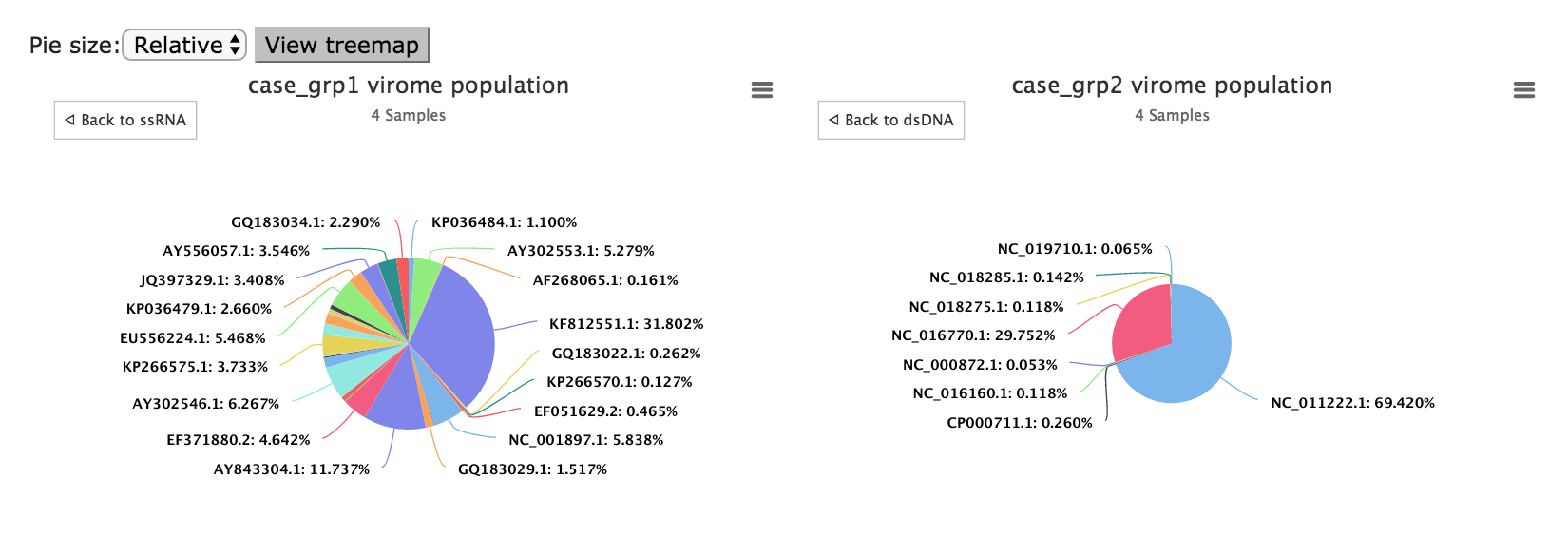


Figure 8 Diversity plots are interactive maps. The top row (A) shows the viral diversity at the DNA/RNA group level while bottom row (B) shows the pie charts interactively clicked down to accession level. The colors and sizes indicate relative viral populations and clicking on the maps imitates drilling down the relevant viral taxonomy levels.

## Summary Boxplots

Within the Summary and diversity panel, population summary information and diversity, calculated as Shannon index, are calculated and then plotted, the groups, when defined, are compared as shown in figure 9 below. Diversity comparisons, statistics and particularly their interpretation are highly complex and variable within the context of the experiment. We recommend that investigators used the raw results from downloads and assess statistic methods and metrics based on literature and data distribution.


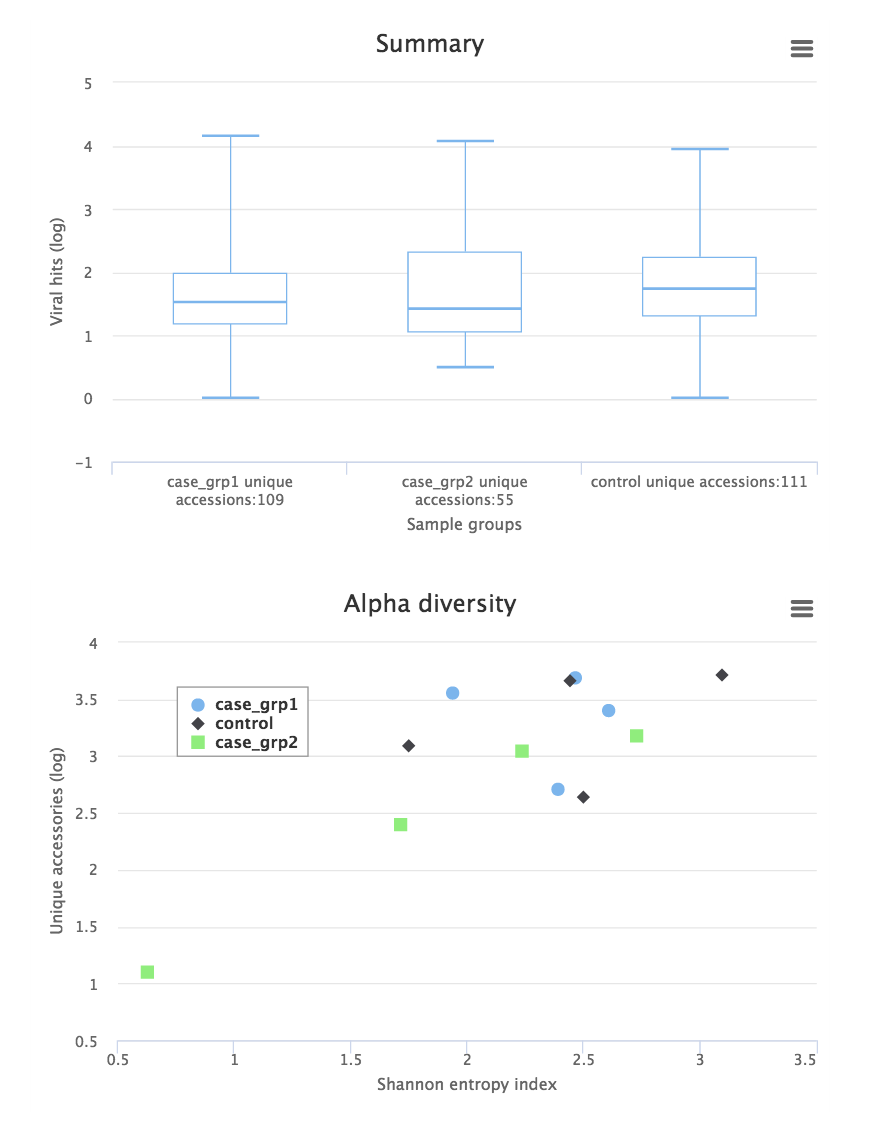


Figure 9 Summary box and diversity plots grouped or labeled with relevant sample assignment groups defined in the optional mapping file. Unassigned is the label used when no groups are defined.

## Sample Viral Matched table

Another key component of the Vipie results is the matched table where investigators can view and sort virus hits to the input samples, much like Excel spreadsheet. As shown in Figure 10 below, the Viral hits table displays the sample (columns) hits to viral accession ids (rows) where the viral accession labels include id, description, and taxonomy levels taken from GenBank from NCBI. After the taxonomy level, the columns are labeled with the sample names together with a percentage calculated from sample hits divided from a normalized total of 100,000. The red intensity background of the data cells reflects the increase hit size. Users can custom select the number of rows shown on the page. An example is partially shown in the figure below. As demonstrated in Figure 10B searching for Human rotavirus, flexible search is built in for string search across all cells, and the columns are all sortable, in both directions.

A
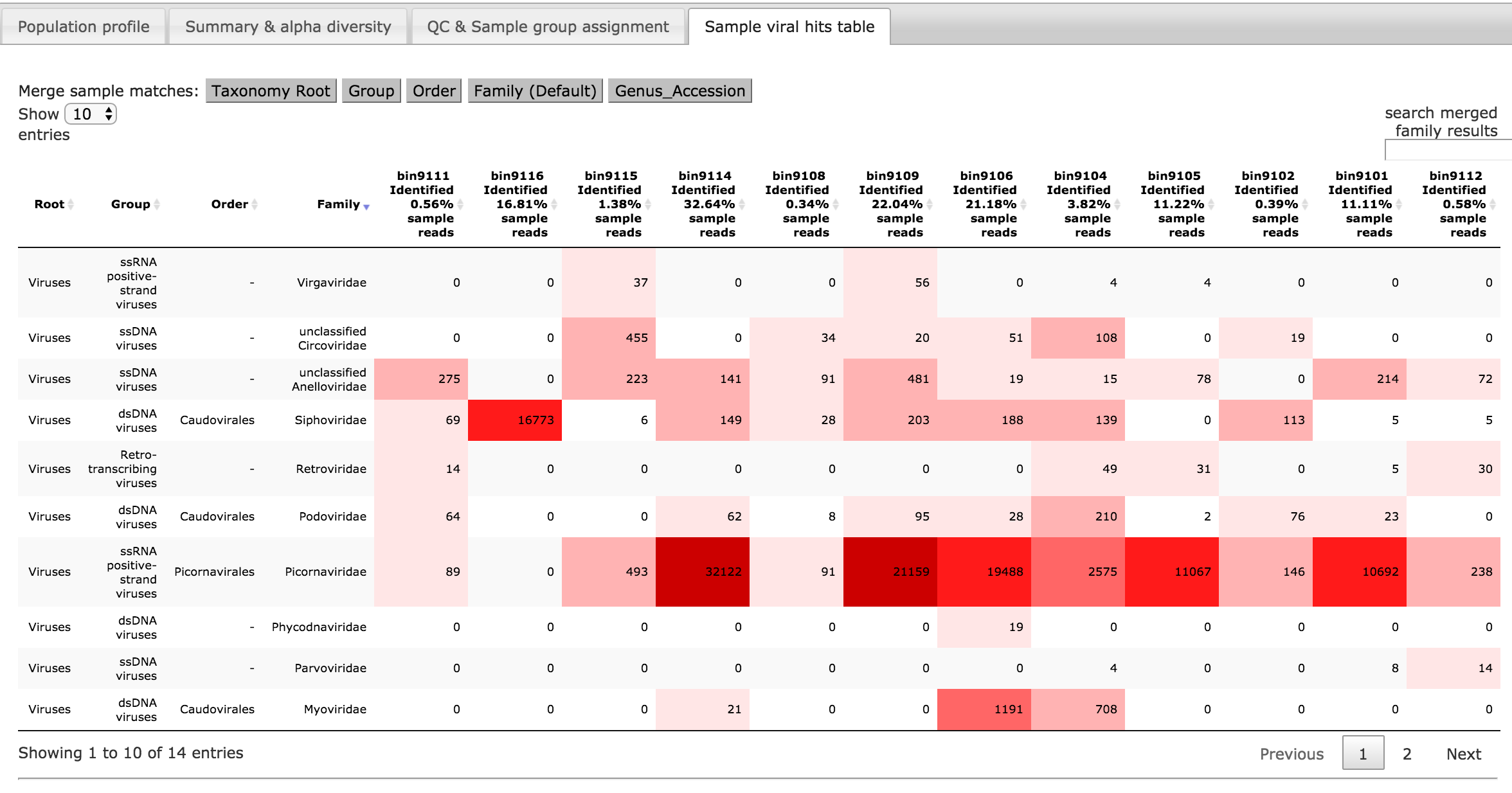


B


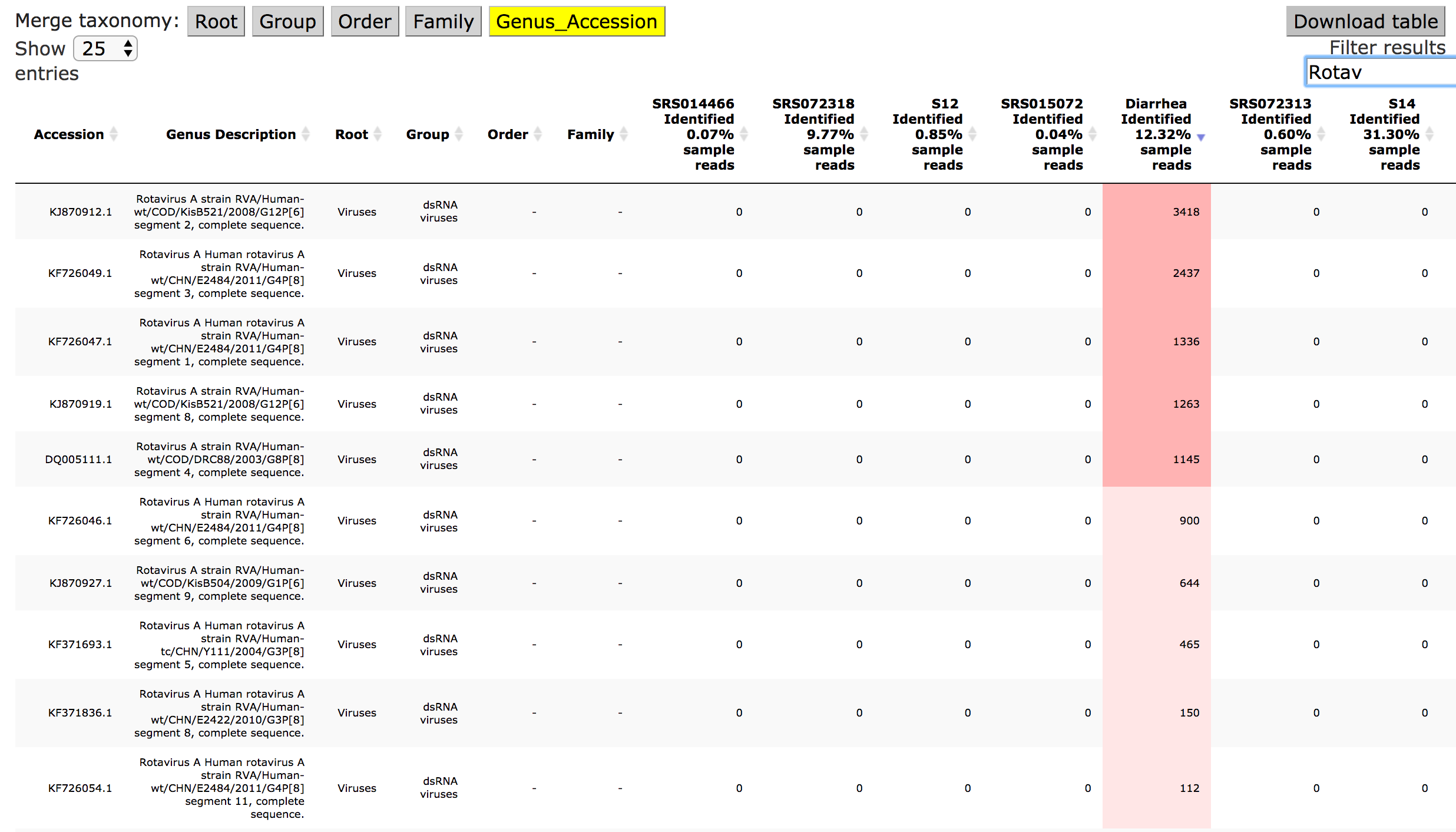


Figure 10 Web based table allowing dynamic taxonomy merging, search and sortable columns. A) The raw table is available as part of the results download. The increasing red color background indicates hit sums of greater than 100,1000 and then 10000. B) Table supports subsearch and sorting as demonstrated searching for ‘Rotav’ and sorting on Diarrheal sample.

## Clustered heatmaps

## The sample viral hit table is essentially a numeric matrix and such data structures can be clustered, where the inter-sample (columns) distances are computed on the viral hit similarities. Figure 11 is downloaded from results.

##

Figure 11 The downloaded pdf also contains a R generated clustered heatmap. Group reassignment and re-plotting of new heatmaps, can be done dynamically within web results.

## Mapping assignment post analysis

Sample assignment functionality is allowed on all samples whether they have been assigned or not to existing groups. The figure below uses the same 13 samples set and the existing control group samples are manually reassigned to case_grp1. File upload is also supported. The before and after population maps are shown in figure 12 below. It is expected that the population map size of case_grp1 is more than double of case_grp2 and also note that sample 11 is not eligible for assignment as the contigs produced is too low.

| 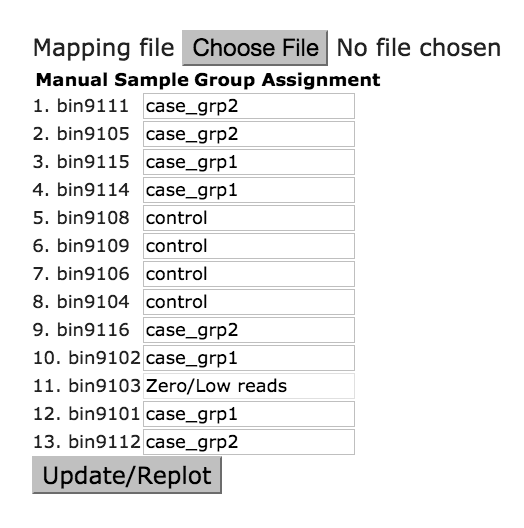 | 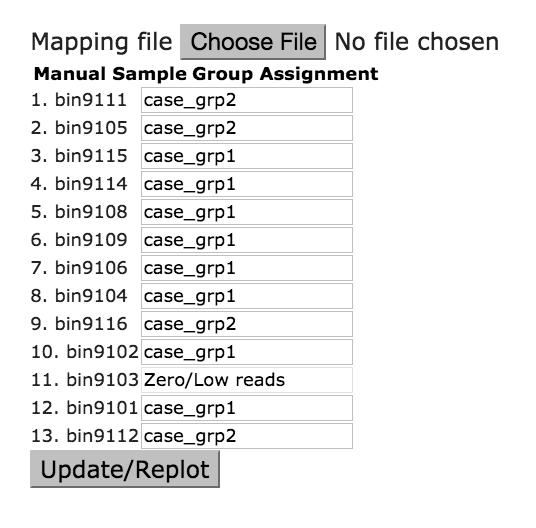 |
| --- | --- |


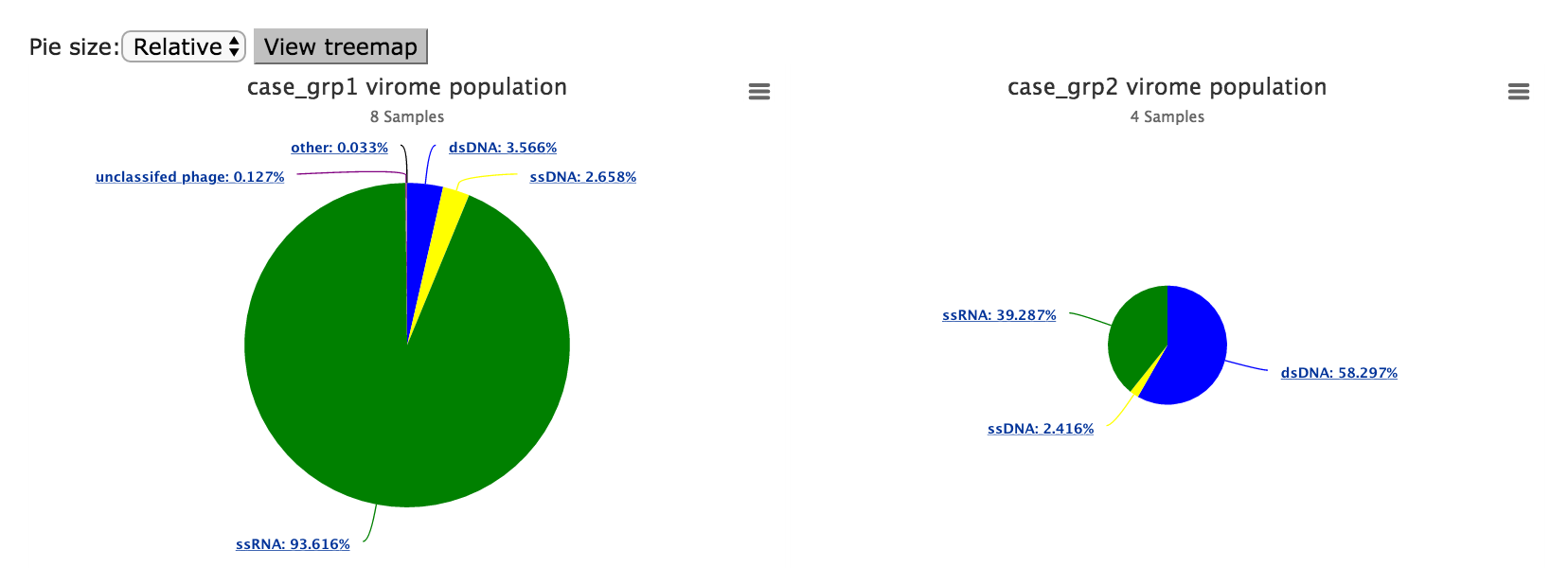


Figure 12 Sample group reassignments dynamically redraw the population plots, including heatmaps.

## QC, Mapping assignment low reads (NA) and Exclude controls

By chance some of the samples/bins associated with FASTQ inputs received low NGS reads (less than 0.5 %), then contigs cannot be assembled. These samples cannot be assigned due to low quality and subsequent low contigs from assembly. Sample 9103 from the test input is an example and also confirmed in the QC line plots of figure 13 showing the number of reads following QC steps.


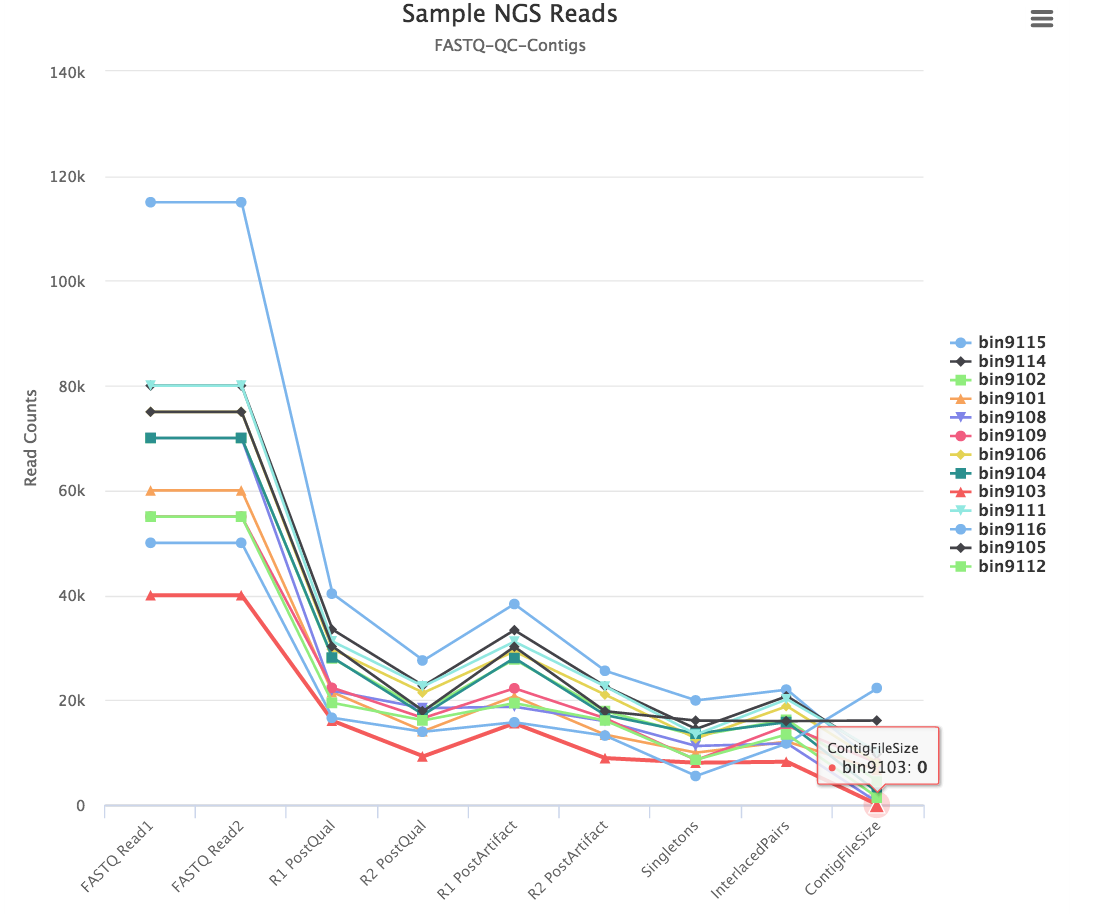


Figure 13 QC plot showing the number of input sample reads to contig sizes following quality control steps.

On the other hand, investigators have the option of excluding samples, for example, serving as positive controls. This is particularly useful on reassignment of R cluster maps as well as the diversity maps. Exclude is available as a drop down and upon selections, click on the Update/Replot function. Selection figure is shown below:

## Viral ‘dark matter’ and distribution of reads

It is frequently reported that a majority of NGS viral reads do not matched known references, hence, the notion of a viral dark matter. The high frequency is due to lack of a common genomic trait, such as 16S for bacterial, and high viral diversity due to its faster rate of evolution. Reads, subsampled from original input up to 1 000 000 are marked as unmapped if they failed to re-map (using bwa-mem and tolerating 2 mismatches per 100 bases) to any of the ‘best’ accessions obtained from BLAST operations using assembled contigs. They are reported as dark matter after removal of human sequences (build 37/hg19) and known bacterial ribosomes, including 16S and 23S. The results and raw files are accessible from the web results page and Figure 14 below portraits the report and also a build in interactive feature.

|  |  |
| --- | --- |

Figure 14 Stacked bar showing distribution of reads. The y axis represents the number of reads while the samples are plotted on the x axis. The dark viral matter, represented in black, dominates the distribution. Red is known viral hits, and blue is ribosome, with green for matches to human reference. The right plot has viral dark matter turned off where sample reads proportionally mapped to human are revealed.


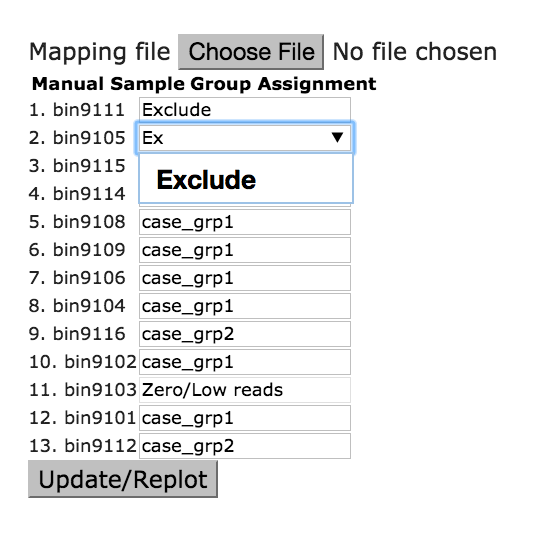


Figure 15 Predefined groups Case, Control and Exclude are provided for easy selection. Researchers can upload xslx map files or also type in any group name. Exclude implies that sample will not be included in the replot.

# User case application – virome population profile results of 11 samples from Human Microbiome Project (HMP), DNA Bank of Japan and in-house Africa metagenomics project.

## Sample protocol preparation

To test the performance of the tool and as part of the manual script; we combined sequencing data from 8 HMP samples, 1 Japanese sample used in VirusTap (<https://gph.niid.go.jp/cgi-bin/virustap/index.cgi>) validation, and finally 2 samples from unpublished African experiment. To be specific, the 1.2 GBs archived consisted of almost 30 million reads and end to end parallel processing took 82 minutes. Execution time is dependent on file and sample sizes, sample read depth, server load and network speed. Vipie is currently deployed on a Linux server (Intel(R) Xeon(R) CPU E5-2630 0 @ 2.30GHz) with 24 CPUs and 64 GBs of memory.

The NGS reads are produced from Illumina, though from different generation machines and enrichment chemicals. Vipie is agnostic to machine software image and sample prep. While these are important metadata, as long as paired end fastq files are de-multiplex (following naming conventions from section 1), they can be used and Vipie QC metrics will report usable reads. A smaller subsampled (20% random subsampled, 225 Megabytes) zip file is available here:

https://binf.uta.fi/vipie/data/vipie_archive_ssampled.zip

The diarrheal sample from DNA Bank of Japan was found to have high human Rotavirus content, 14 different accessions amounting to ~10K hits. This mostly concurs with VirusTAP findings though VirusTAP had fewer accessions, 7, as it only reports contig mappings and also filters known references prior to scoring and rules out viral mimicry discovery.

## Parameters


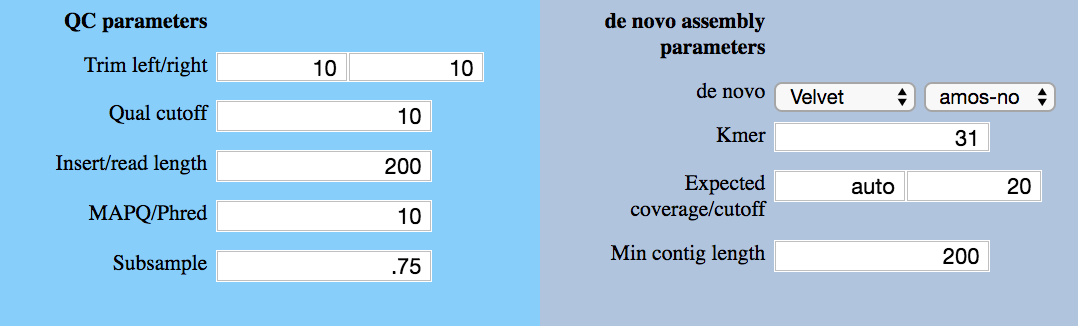


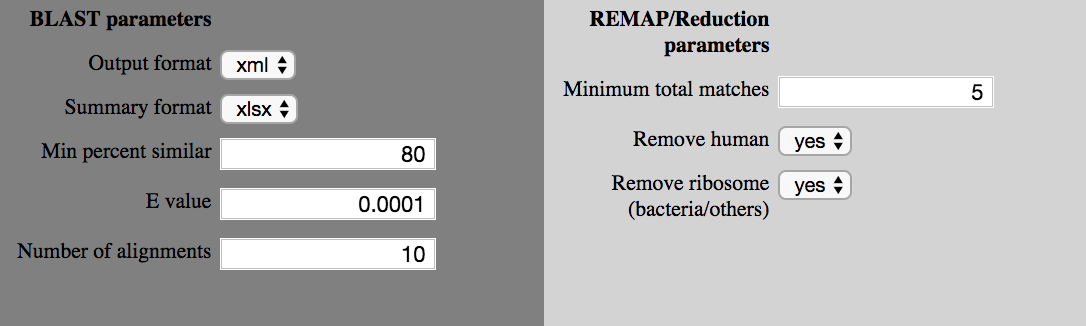


Figure 16 Parameters used for analysis. Kmer was set to 31 as HMP samples, sequenced with older machines, had shorter read lengths.

## Results

As indicated in our manuscript, interactive results are available here:

<https://binf.uta.fi/vipie/results.html?key=DeNxaqSIdG>

Please note that all parts of the data presented may not be used, shared or cited without explicit permission from Heikki Hyöty of University of Tampere. The result figures below indicate sample categories reassigned after uploading this mapping file (https://binf.uta.fi/vipie/data/mapping.tsv).

## Custom sample group maps


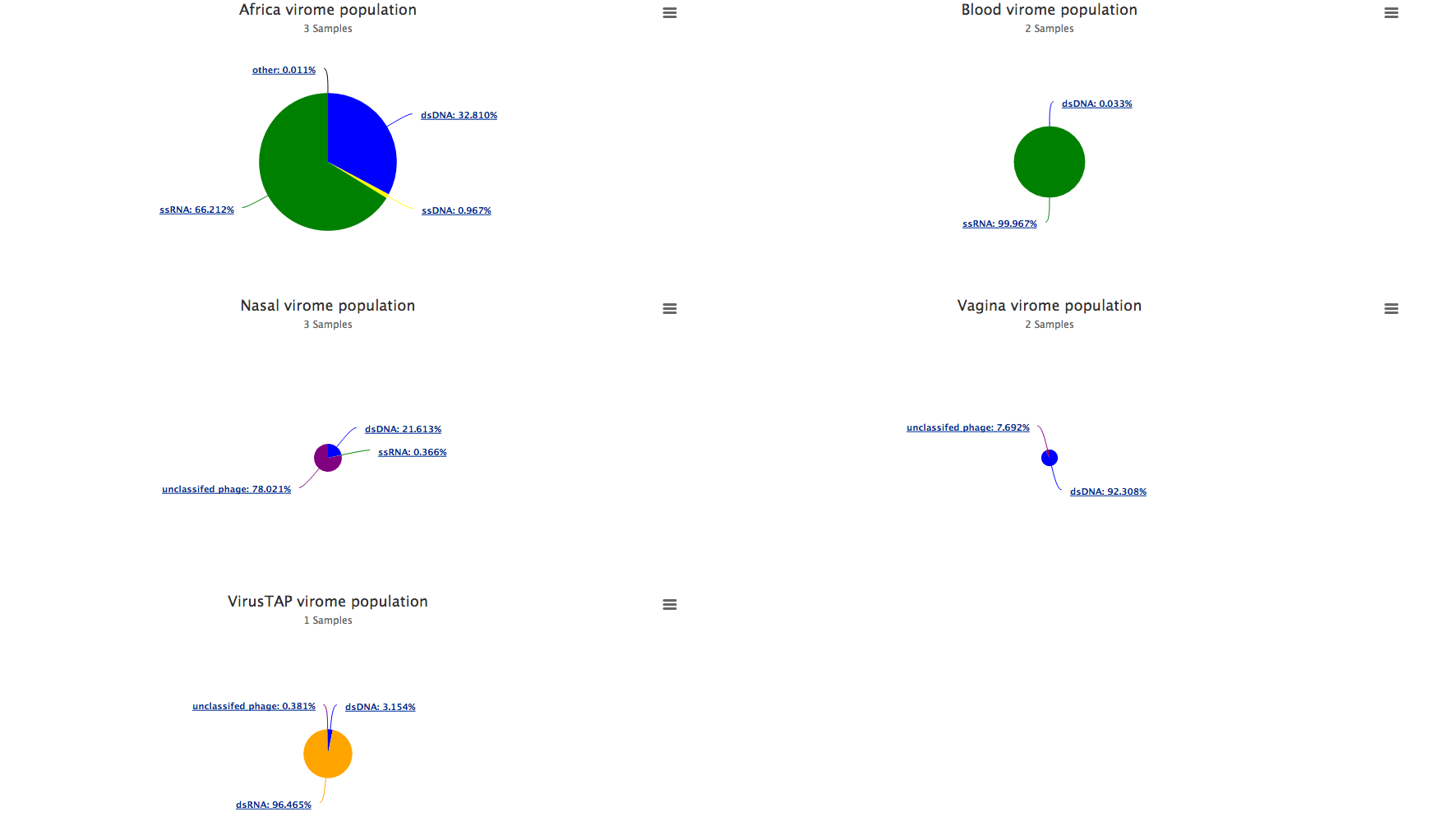


Figure 15 Vipie results of 11 NGS samples covering 5 assignments representing Japan diarrheal (1), African (3) stool and 3 HMP sample types – blood (2), nasal (3), vagina (2). 156 unique accessions were found and African samples had the highest percentages, in uniqueness and quantity. The pie chart sizes are relative to size, and HMP nasal and vagina samples had low hits, averaging about 6 accessions, which is in lined with previous reports. .

Figure 16 The figure confirms the higher diversity of African samples and also correctly clusters HMP samples together. It is informative that the Diarrheal sample, from gastroenteritis outbreak, had a clear viral signature. Looking at distribution/unmapped report, it also has higher proportion of bacterial ribosome and known viral matches.

# Contact

Vipie is free for nonprofit research and does offer any guarantees. Please contact [jake.lin@uta.fi](mailto:jake.lin@uta.fi) and [ondrej.cinek@lfmotol.cuni.cz](mailto:ondrej.cinek@lfmotol.cuni.cz) if you have any questions.
